# Ginsenosides Rb3 and Rd reduce polyps formation while reinstate the dysbiotic gut microbiota and the intestinal microenvironment in Apc^Min/+^ mice

**DOI:** 10.1038/s41598-017-12644-5

**Published:** 2017-10-02

**Authors:** Guoxin Huang, Imran Khan, Xiaoang Li, Lei Chen, Waikit Leong, Leung Tsun Ho, W. L. Wendy Hsiao

**Affiliations:** 1 State Key Laboratory of Quality Research in Chinese Medicine, Macau University of Science and Technology, Macau, China; 2 Department of Pathology, University Hospital, Macau University of Science and Technology, Macau, China; 3 Department of Genetics, Rutgers University, New Brunswick, USA

## Abstract

Studies showed that manipulation of gut microbiota (GM) composition through the treatment of prebiotics could be a novel preventive measure against colorectal cancer (CRC) development. In this study, for the first time, we assessed the non-toxic doses of the triterpene saponins (ginsenoside-Rb3 and ginsenoside-Rd) – as prebiotics – that effectively reinstated the dysbiotic-gut microbial composition and intestinal microenvironment in an Apc^Min/+^ mice model. Rb3 and Rd effectively reduced the size and the number of the polyps that accompanied with the downregulation of oncogenic signaling molecules (iNOS, STAT3/pSTAT3, Src/pSrc). Both the compounds improved the gut epithelium by promoting goblet and Paneth cells population and reinstating the E-cadherin and N-Cadherin expression. Mucosal immunity remodeled with increased in anti-inflammatory cytokines and reduced in pro-inflammatory cytokines in treated mice. All these changes were correlating with the promoted growth of beneficial bacteria such as *Bifidobacterium* spp., *Lactobacillus* spp., *Bacteroides acidifaciens*, and *Bacteroides xylanisolvens*. Whereas, the abundance of cancer cachexia associated bacteria, such as *Dysgonomonas* spp. and *Helicobacter* spp., was profoundly lower in Rb3/Rd-treated mice. In conclusion, ginsenosides Rb3 and Rd exerted anti-cancer effects by holistically reinstating mucosal architecture, improving mucosal immunity, promoting beneficial bacteria, and down-regulating cancer-cachexia associated bacteria.

## Introduction

CRC is ranked second among all the cancer incidences worldwide. Among USA population, approximately 4.3 percent of men and woman are diagnosed for colon and rectum cancer at some point during their lifetime^1^. Epidemiological and animal studies have indicated that western high fat diet is believed to be the key risk factor responsible for the high incidence of CRC^2,3^. This observation was supported by the recent findings in which high animal fat diet altered the gut microbial composition, leading to microbial dysbiosis and inflamed gut epithelium^4–6^. Indeed, being an integral part of the host digestion system and in direct contact with tumors, gut microbiota (GM) has been recently focused for its defined role in CRC progression^7–9^. Generally, CRC patients are characterized with lower GM diversity and reduced abundance of beneficial bacteria^10^. In addition, some bacterial species have been identified for contributing to CRC development. Some of these well-known bacteria include *Fusobacterium spp*., *Streptococcus bovis*, *Bacteroides fragilis*, *Peptostreptococcus spp*., and *Porphyromonas* spp.^8–12^.

On the other hand, studies have shown that the microbial dysbiosis can be reversed by the combined treatments of functional food, prebiotics, and/or probiotics^13,14^. For instance, a resveratrol-supplemented diet significantly reduced the colonic tumors in rats through the reduction of bacterial glucuronidase^15^. Coffee phenolic phytochemical was found to suppress colon cancer metastasis by targeting protein kinases, particularly MEK and TOPK^16^. Strategy of combined treatments with beneficial microbes and specific dietary compounds was found to prevent the development of colonic polyps in both animals and humans^17^. Overall, the dynamic interplay between the GM and the ingested dietary compounds impacts on cancer risk and treatment. For this reason, probiotics- and prebiotics-based GM modifications have been proposed as an alternative approach for the treatment and prevention of CRC^18^. However, broad spectrum benefits of these therapies are still unclear and discrepancies exist^19,20^.

Our previous studies have indicated that triterpenoid saponins extracted from a medicinal plant *Gynostemma pentaphyllum* (Gp) can enhance beneficial intestinal bacteria and exerted prebiotics-like effects to the treated mice^21^. We also showed that Gp saponins (GpS) significantly reduced tumors growth and count in both nude mice and Apc^Min/+^ mice^21,22^. However, the bioactive phytocompounds of GpS are unknown. Thus, in this study, two purified compounds (gypenoside Rb3 and gypenoside Rd) of Gp were investigated for the cancer protective effects and the impact on GM in the Apc^Min/+^ colorectal cancer mouse model. Both Rb3 and Rd exhibited anti-cancer effect in our cellular model (unpublished data). The mice were daily administered with Rb3 or Rd for eight weeks. At the end of the treatments, fecal samples were collected and evaluated for changes in GM diversity and composition. Immunohistochemistry, qRT-PCR and Western blot were employed to determine changes in mucosal cytokines and intestinal architecture.

## Results

### Rb3/Rd reduced the intestinal polyps without affecting the body weight, food and water consumption of Apc^Min/+^ mice

**Figure 1:**
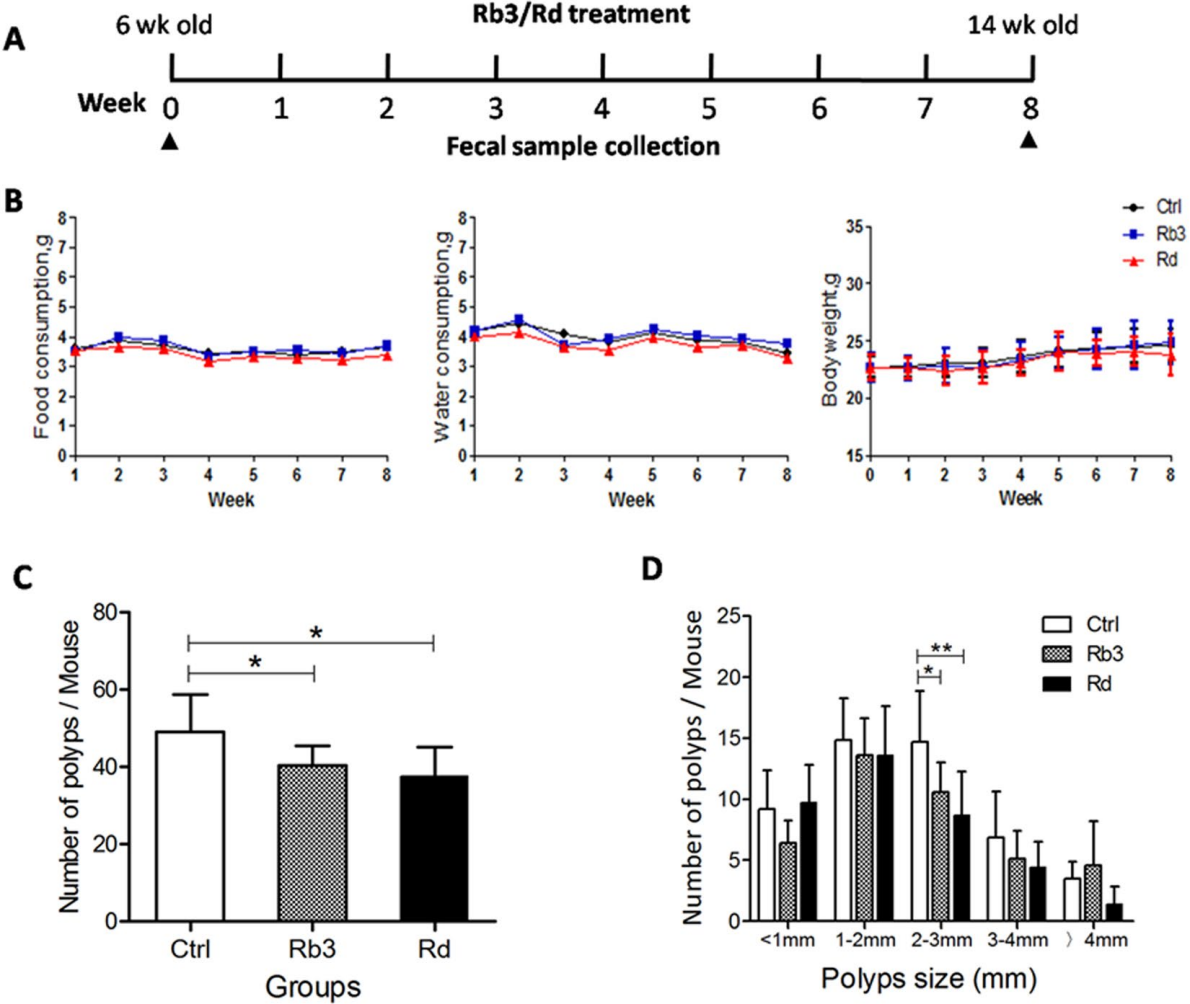
Effects of ginsenosides Rb3/Rd on the intestinal polyp formation in Apc^Min/+^ mice. (**A**) Schematic diagram of experimental design. (**B**) The profiles of body weight, diet and water consumption. (**C**) Effect of Rb3/Rd on total number of polyps. (**D**) The distribution of different diameter of polyps. Data is presented as the mean ± SD; n = 7/group. ∗p ≤ 0.05: ∗∗p ≤ 0.01.

Six-weeks-old mice were subjected to Rb3 and Rd treatment, before the appearance of the intestinal polyps. The treatment scheme is shown in Fig. 1A. All the mice were monitored for food intake, water consumption, and weight changes.Throughout the experiment, we did not observe Rb3/Rd-associated weight loss in mice (Fig. 1B). In addition, none of the treated mice showed variations in food and water consumption (Fig. 1B). Whereas, the number and size of the polyps were effectively reduced by Rb3/Rd treatments (Fig. 1C). Comparatively, the anti-tumor property of Rd was more apparent than Rb3.

### Rb3/Rd improved the epithelial barrier of Apc^Min/+^ mice

**Figure 2:**
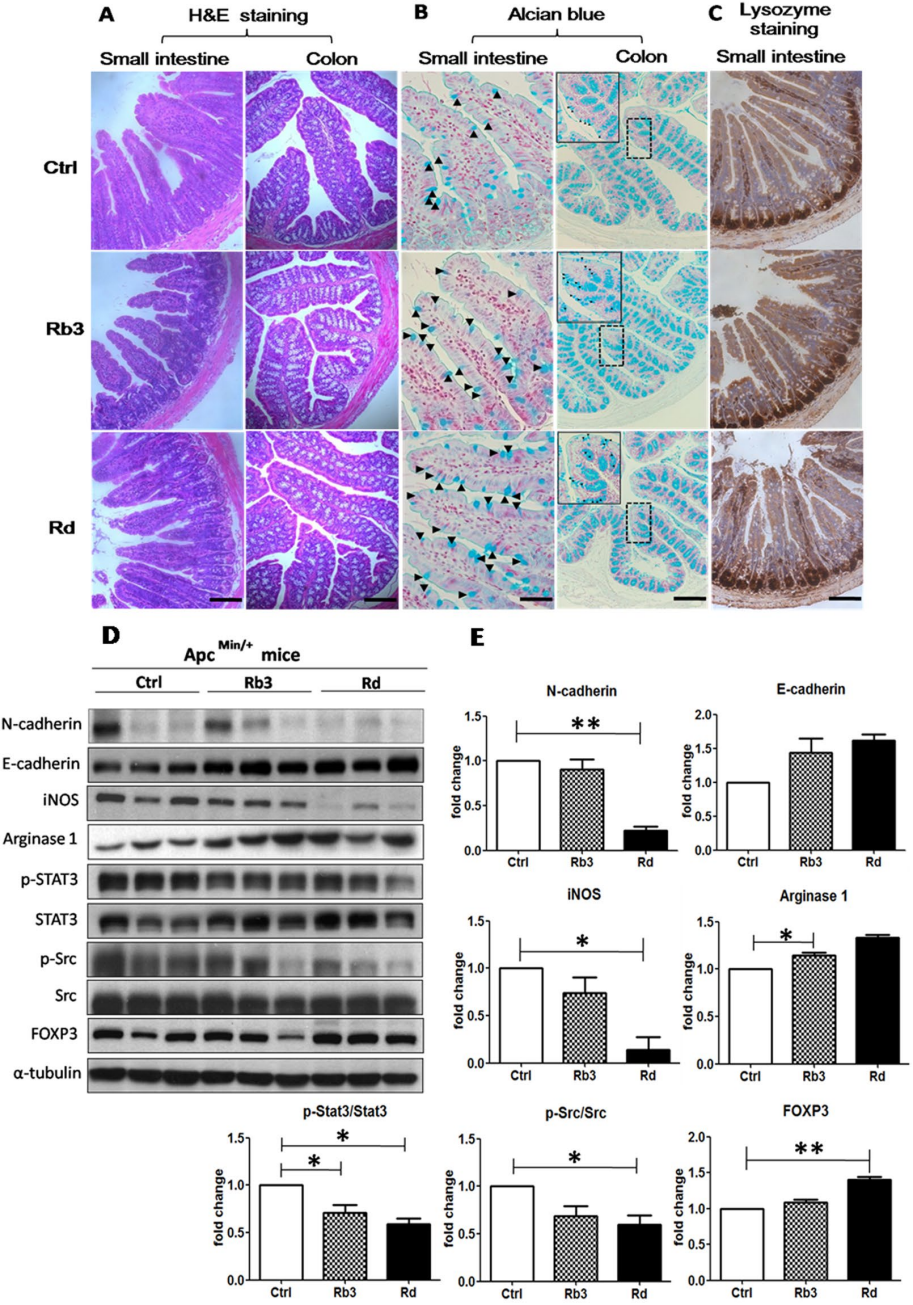
Effects of Rb3/Rd on the intestinal epithelium of Apc^Min/+^ mice. (**A**) H&E staining of the formalin-fixed sections of small intestine and colon. (**B**) Alcian blue staining of goblet cells. Arrows indicates the presence of goblet cells that distribute along the epithelial layers of the small intestine and the colon. (**C**) IHC staining of Paneth cells with anti-lysozyme antibody. The dark brown staining at the bottom of the crypts indicates the location of the Paneth cells. (Scale bars, 100 μm). (**D**) Western blotting analysis of mucosal biomarkers with the indicated specific antibodies. The figure is developed from various blot gels. The full-length Western blot gels are shown in supplementary file Fig. S5 (**E**) Fold change of protein expression. The α-tubulin was used as a loading control. Each lane represents sample obtained from individual mouse (n = 3). Data is presented as the mean ± SEM. Statistical analysis was performed with one-way ANOVA. ∗p ≤ 0.05: ∗∗p ≤ 0.01.

Apc^Min/+^ mice develop polyps in small intestine and in the colon that cause gut epithelial dysfunction^23^. In order to observe the gut epithelial integrity after Rb3 and Rd treatments, we examined the epithelial morphology, goblet cells, and Paneth cells of the experimental mice. Based on the hematoxylin and eosin (H&E) staining, treatments with Rb3/Rd did not alter the gut epithelial general morphology compared to the control group (Fig. 2A). However, a substantial increase in goblet cells count (visualised with Alcian blue stain) was noticed in the small intestine and colon of the Rb3/Rd treated mice (Fig. 2B). Moreover, lysozyme staining indicated a significant increase in Paneth cell count in the small intestine of Rb3/Rd treated mice (Fig. 2C). Both goblet cells and Paneth cells are the main cell types in the epithelial layer and play an important antimicrobial role to protect intestinal tract from the invasion of foreign pathogenic microbes.

### Rb3/Rd downregulated the expression of biomarkers associated with the risk of CRC development

E-cadherin is crucial for cellular adhesion and also participate in Paneth cells maturations^24^. Overexpression of N-cadherin compared to E-cadherin has been associated with CRC progression^25^. Here, we observed that Rb3/Rd treatments improved epithelial architecture by up-regulating E-cadherin and down-regulating N-cadherin expression (Fig. 2D,E). We also confirmed that the expression of phosphorylated signal transducer and activator of transcription 3 (p-STAT3) was reduced in the colonic mucosa of Rb3/Rd treated mice (Fig. 2D,E). STAT3 is one of the main targets for cancer therapies. Its activation could upregulate N-cadherin meanwhile downregulate E-cadherin^25^. Suppression of p-Src and iNOS proteins were also observed in mice treated with Rb3 and Rd. On the other hand, FOXP3 and arginase-1 were upregulated upon Rb3 and Rd treatments. Overall, Rd exerted stronger impact than Rb3 on the expression of oncogenic genes displayed in Fig. 2.

### Rb3/Rd suppressed pro-inflammatory cytokines and enhanced anti-inflammatory cytokines

**Figure 3:**
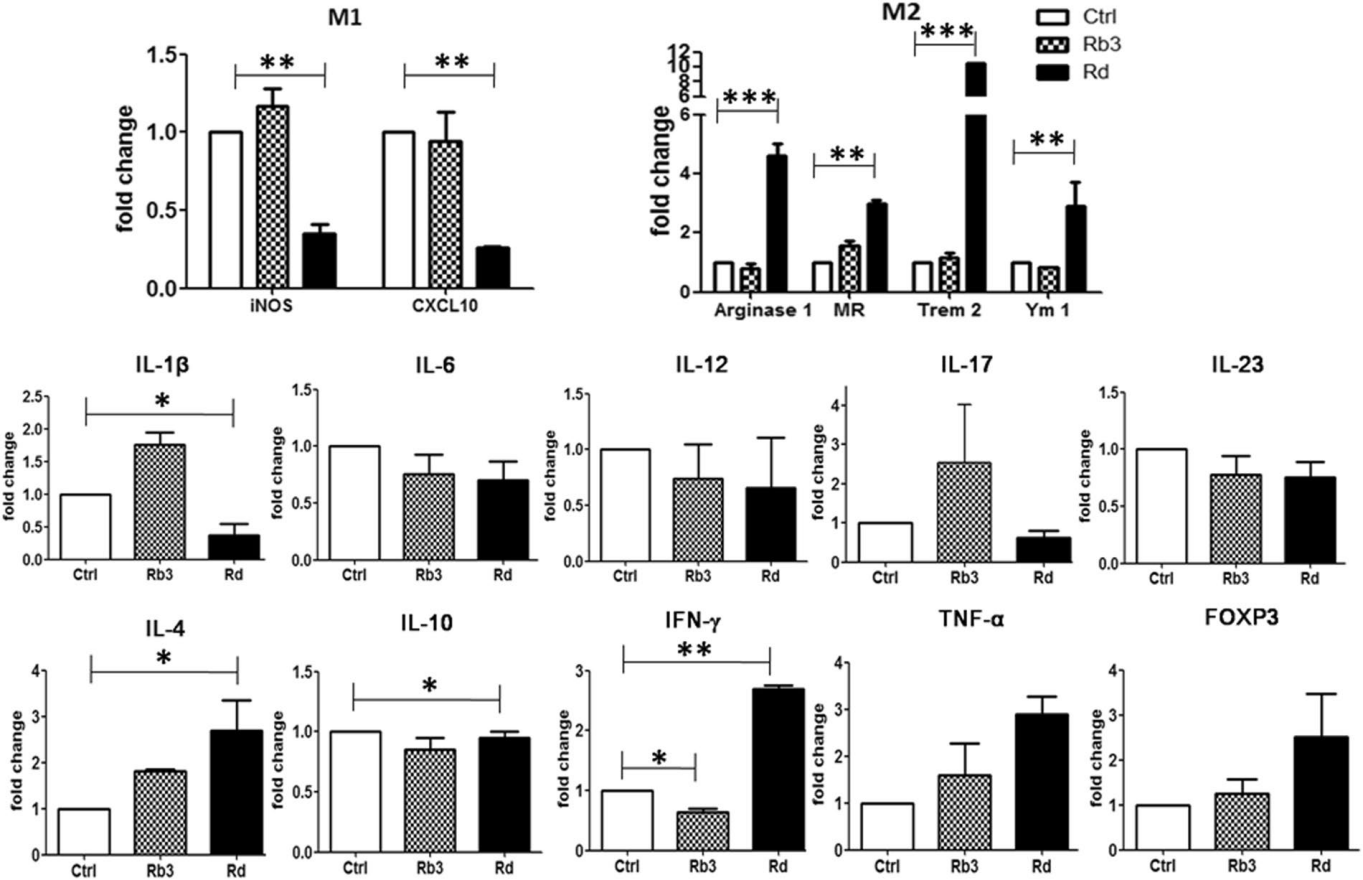
Rb3/Rd modulated transcriptional expressions of mucosal cytokines. Data was normalized to β-actin, and expressed as fold change relative to control group. Data is presented as the mean ± SD; n = 3/group. ∗p ≤ 0.05; ∗∗p ≤ 0.01.

It is known that individual members of the intestinal microbiota can markedly alter the inflammatory state of the intestinal immune system^26^. Inflammatory mediators, particularly cytokines, are the key determinants to check the pro- and anti-inflammaotry status of the gut epithelial microenvironment^27^. In this study, qRT-PCR was employed to evaluate the expression profile of cytokines in the mucosa of the small intestine. Contrary to Rb3, Rd treatment markedly (p < 0.01) decreased the expression profiles of iNOS and CXCL10 (Fig. 3). Both genes encoded the main effector molecules that are associated with pro-inflammatory M1 phenotype^28,29^. Contrary to Rb3 treatment, we noticed that Rd treatment upregulated mRNA expression of M2 macrophage phenotypic markers such as arginase-1, MR, Trem-2 and Ym-1 compared to control group (Fig. 3). These biomarkers possess anti-inflammatory properties^24^. The level of other pro-inflammatory cytokines, such as IL-1β, IL-6, IL-12, IL-17, and IL-23, were profoundly lower in Rd-treated mice compared to Rb3-treated mice (Fig. 3). Unexpectedly, we noticed the elevation of IL-1β and IL-17 in Rb3-treated mice compared to the control group (Fig. 3).

Enriched expressions of TNF-α and FOXP3 were observed in Rb3/Rd treated mice compared to the control group. The abundance of IL-4 and IL-10 were significantly (p < 0.05) higher in Rd-treated mice compared to control group (Fig. 3). IL-4 and IL-10 are involved in M2 macrophage polarization that subsequently induces tissue repair and initiates anti-inflammation^24^.

### Rb3/Rd altered GM composition and enriched GM diversity

**Figure 4:**
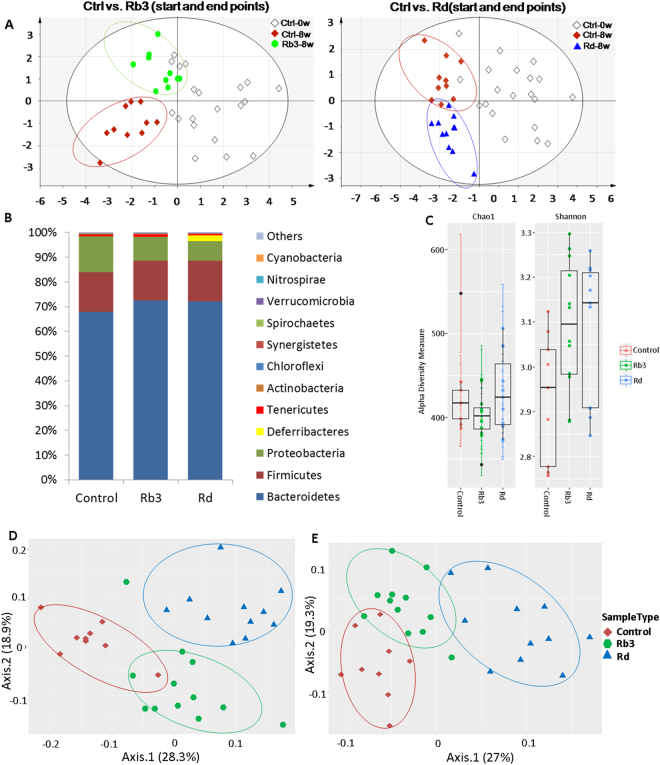
Rb3/Rd treatments altered GM composition and enriched GM diversity. Fecal DNA samples were obtained from experimental animals as described in Fig. 1(A). Comparative ERIC-PCR data analysis using PLS-DA plot. (**B**) Average relative abundance of the dominant bacterial phyla. The *y*-axis displays the average percentages of sequences reads. “Others” indicates minor phyla with abundance less than 0.03% per each phylum. The color of steric represents significant changes in abundance in the respective group. (**C**) Alpha diversity analysis. (**D**) GM clustered using PCoA of the un-weighted UniFrac matrix. (**E**) GM clustered using PCoA of the weighted UniFrac matrix. The percentage of variation explained by the principal coordinates is indicated on the axes. Each dot corresponds to individual mouse of the community of control, Rb3 and Rd groups. ∗p ≤ 0.05; ∗∗p ≤ 0.01.

**Figure 5:**
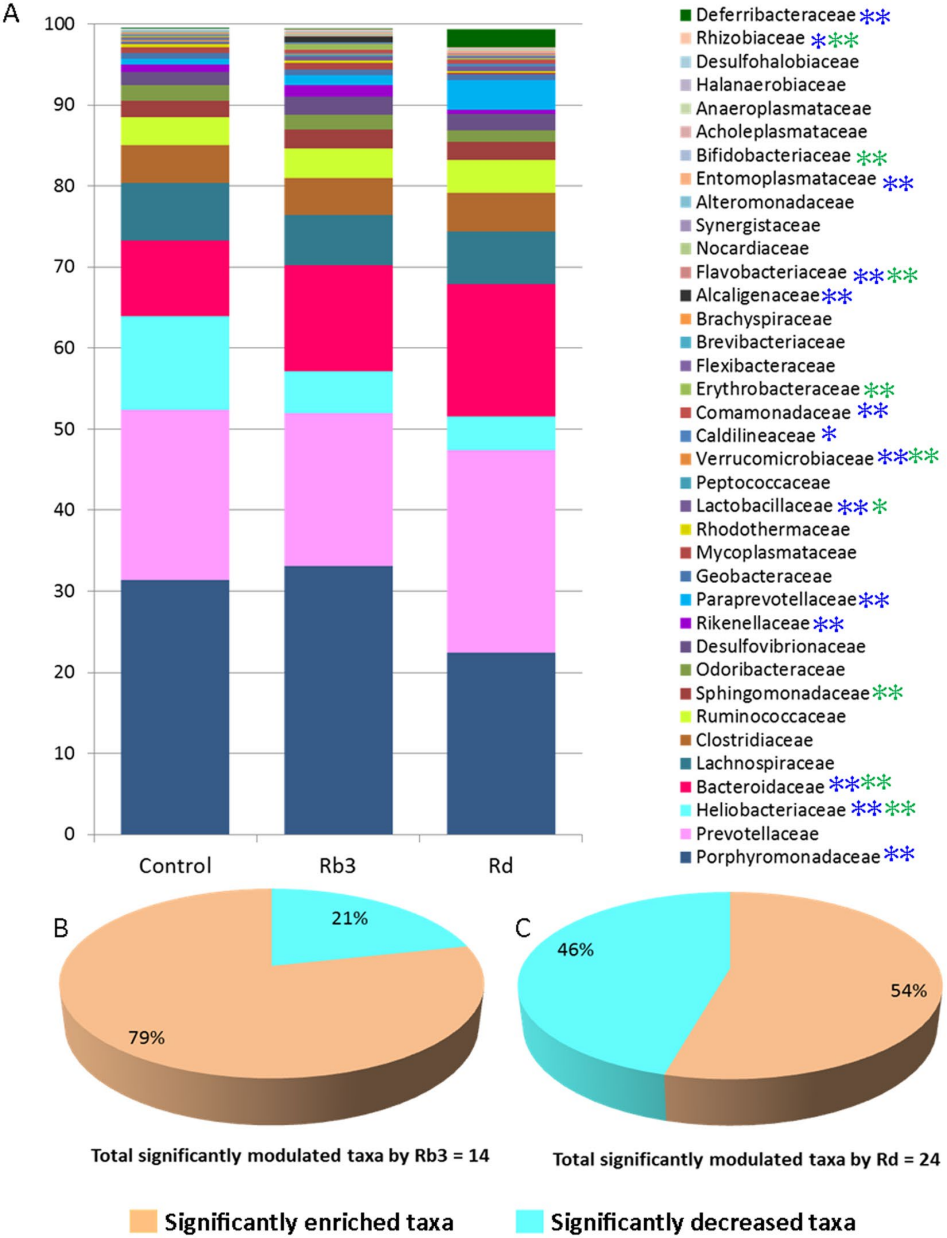
Graphical presentation of dominant families in the control and Rb3/Rd treated APC^Min/+^ mice. (**A**) Average relative abundance of dominant families. The cutoff point was set to 0.05% average relative abundance. The *y*-axis displays the average percentages of sequences reads. The highest number of significant changes in families was observed in Rd treated group. Among the significantly-modulated taxa, Rd effect was most profound on class Bacteroidia in term of OTUs number and their abundance. The stearic color is representing significant change in respective group compared to control group – as green color is indicating Rb3 and dark-blue color is indicating Rd group (**B**) Showing average relative abundance of all families that significantly enriched or declined with Rb3 treatment. (**C**) Showing average relative abundance of all families that significantly enriched or declined with Rd treatment. The color of steric represents significant changes in abundance in the respective group. ∗p ≤ 0.05: ∗∗p ≤ 0.01.

The Rb3/Rd-induced changes in microbial composition were first evaluated by analyzing the fecal DNA samples using the ERIC-PCR method. We observed differential modulation patterns in Rb3- and Rd-treated groups compared to the 0-day and 8-week control groups (Fig. 4A). For in-depth analysis, 16S RNA gene pyrosequencing was employed to evaluate the impact of Rb3/Rd on GM diversity and composition. In total, around 23 phyla,184 families, 386 genera and 731 species were detected. Out of 23 detected phyla, majority of the sequence reads were identified for Bacteriodetes, Firmicutes, Proteobacteria, and Tenericutes (Fig. 4B). Compared to the Rb3 and control groups, higher diversity (Chao1>420 and Shannon>3.1) were recorded in the Rd-treated group (Fig. 4C). Moreover, using UniFrac distance analysis (weighted and unweighted), clear shifts in bacterial composition were observed in the Rb3/Rd treated groups compared to control group (Fig. 4D,E). Among the dominant phyla, Rb3/Rd significantly (p < 0.05) down-regulated the relative abundance of phylum Proteobacteria, while increased the Bacteroidetes phylum (Fig. 4B). Beside reduced abundance, we also observed lower species richness related to phylum Proteobacteria (Fig. S1A). Promoted growth of Proteobacteria has been previously associated with inflammation, disturbed mucus layer and CRC progression^30^. Among 184 detected families, the majority of the reads were related to families Porphyromonadaceae, Bacteroidaceae, Helicobacteriaceae, and Paraprevotellaceae (Fig. 5).

Phylogenetic trees at species level were plotted and comparatively analyzed to observe underline changes in the most dominant families such as Porphyromonadaceae (Fig. S2), Prevotellaceae (Fig. S3), and Bacteroidaceae (Fig. S4). Rb3/Rd significantly (p = 0.001) reduced the abundance of Porphyromonadaceae, the most dominantly detected family in control group. This decrease is mainly attributed to the lower abundance of species related to genera *Dysgonomonas*, *Porphyromonas,* and *Parabacteroidetes* (Fig. S2). Contrary, Prevotellaceae enriched with Rd treatment (Fig. S3) that could be mainly attributed to the promoted growth of species from genera *Prevotella* and *Paraprevotella* (Fig. S3). Moreover, family Bacteroidaceae significantly (p < 0.01) enriched with Rb3/Rd treatments. After annotating species from family Bacteroidaceae, we observed the promoted growth of several health-promoting bacteria such as *Bacteroides vulgatus*, *Bacteroides xylanisolvens*, *Bacteroides gallinarum*, and *Bacteroides acidifaciens* (Fig. S4).

**Figure 6:**
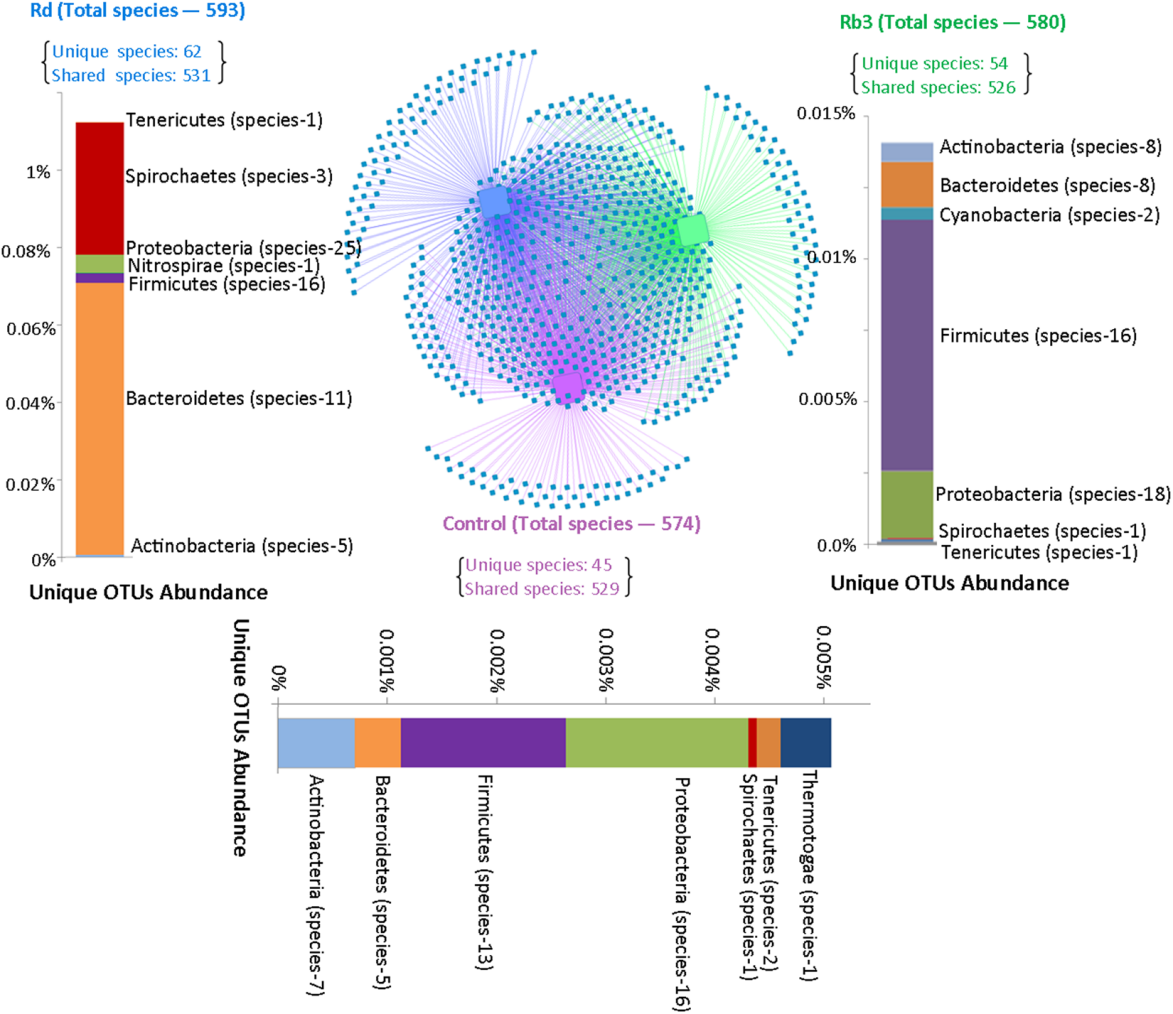
Cytoscape network representation of total, shared and unique species in the control and Rb3/Rd treated APC^Min/+^ mice. Shared species are represented as inter-connected strokes and unique species are shown as outward strokes – uniquely present to each group. The count of total, shared and unique species are mentioned in each group. Unique species were retrieved, annotated to respective phyla and presented in bar-plot under each group name. The number of unique species related to each phylum is mentioned in parenthesis. Scale is showing relative percentage abundance. In control group, phylum Proteobacteria is the most abundance and diverse unique phylum (with 16 unique species). In Rb3 group, Proteobacteria is the most diverse phylum (with 18 unique species), however, phylum Firmicutes is abundant. Similarly in the Rd treated group, Bacteroidetes is the most abundant phylum whereas Proteobacteria is the most diverse phylum (with 25 unique species) in term of unique species.

Using Cytoscape networking, a total of 161 unique species were identified among the three groups. The number of total detected species was slightly higher in Rd-treated (593) and Rb3-treated (580) groups compared to control group (574) (Fig. 6). The unique species detected in the Rd-treated group constituted >1% of the total OTUs in Rd group. About 0.015% and 0.005% of Rb3 and control were comprised of unique species, respectively (Fig. 6). The majority of the unique species were related to phyla Bacteriotedes, Firmicutes, and Proteobacteria (Tables S1, S2 and S3). Some of the unique species inhabiting APC^Min/+^ mice disappeared with Rb3/Rd treatments, and has been reported for pathogenicity, inflammation and CRC development e.g. *Acinetobacter junii*, *Bacteroides uniformis*, *Helicobacter winghamensis*, *and Myroides injenensis* ^31–34^. In addition, the majority of unique species in treated groups were belonged to beneficial genera (such as *Lactobacillus*, *Prevotella*, *Bacteroides*) (Tables S2, S3).

### Rb3/Rd treatments promoted the growth of beneficial bacteria

To understand the underline changes at species level, the dataset was first subset to retrieve dominant bacteria with abundance ≥0.01%. Then, the subset was filtered for bacteria with percentile change (≥10%) compared to control. The final list was searched for probiotic properties using PubMed database and presented in Table S4. Among these beneficial bacteria, the well-known probiotic genera such as *Lactobacillus*, *Bifidobacterium*, *Ruminococcus*, *Prevotella*, and *Blautia* were found greatly enriched in Rb3/Rd treated groups (Table S4).

We noticed 252.8% and 63.9% increase in the abundance of *Bifidobacterium choerinum* with Rb3 and Rd treatments, respectively. Similarly, the growth of several *Lactobacillus* species greatly promoted with Rb3 and Rd treatments compare to control group (Table S4). *Bacteroides acidifaciens* is a renowned probiotic species that was abundantly detected in Rb3 (106% increase) and Rd (292% increase) treated groups compared to control group. Several studies have reported the lower abundance of *Desulfovibrio piger* in CRC host^35,36^. And, we observed an enriched abundance of *Desulfovibrio piger* in Rb3/Rd treated groups compared to control group. Moreover, *Bacteroides xylanisolvens*is is another probiotic bacteria that was significantly (p < 0.01) up-regulated with Rb3/Rd treatments^37^. Some of the beneficial species were differentially modulated with Rb3/Rd treatments. For instance, Rd promoted (33.33% increase) while Rb3 down-regulated (9%) growth of *Roseburia faecis* compares to control group. This bacterium is known for butyrate production and antimicrobial peptide production in human gut^38^.

**Figure 7:**
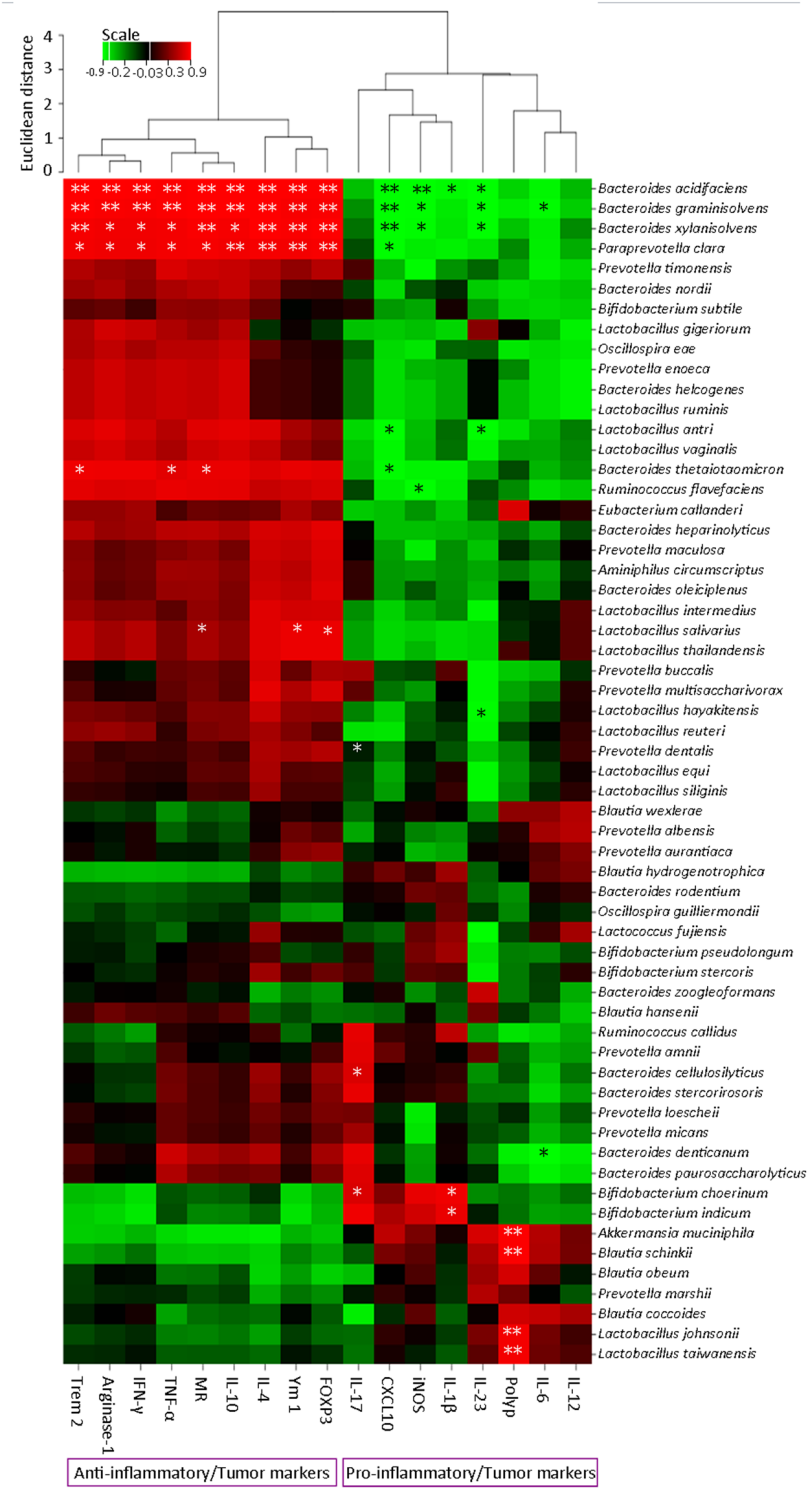
Heatmap presentation of Pearson’s correlation among the beneficial bacterial species and inflammation/tumor-associated markers. Red color is indicating positive correlation; green color is standing for negative correlation whereas black color is showing no correlation. ∗p ≤ 0.05: ∗∗p ≤ 0.01.

We further investigated whether Rb3/Rd-associated improvement in beneficial bacteria is correlating with the expression of tumor-associated markers. Therefore, some of the identified beneficial bacterial species were selected from Table S4. and analyzed for Pearson’s correlation with tumor-associated markers (Fig. 7). Most of the bacteria (for instance *B. xylanisolvens*, *Paraprevotella clara*, *Prevotella timonensis*) were positively relating with anti-inflammatory/tumor markers (e.g. Trem-2, MR, arginase-1, TNF-α) and negatively correlating with pro-inflammatory/tumor markers (e.g. IL-12, IL-6 and iNOS) (Fig. 7). Unexpectedly, some of the benificial species, especially, *Akkermansia muciniphila* (known for anti-obesity properties) and *Blautia* species (that are commensal butyrate-producers)^38^, were positively correlating with pro-inflammatory/tumor markers (Fig. 7).

### Rb3/Rd treatments down-regulated cachexia-associated bacteria

**Figure 8:**
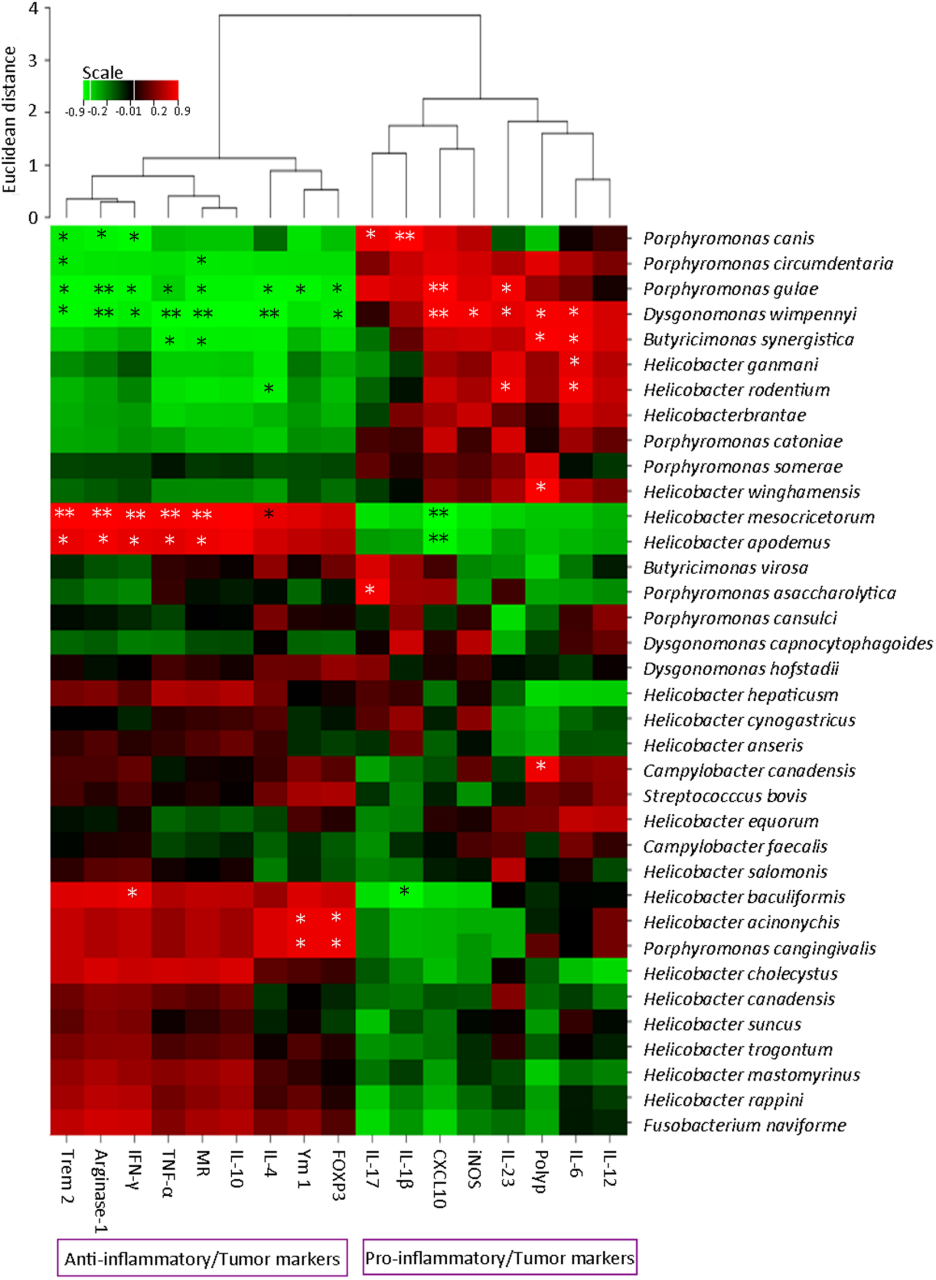
Heatmap presentation of Pearson’s correlation among the cachexia-associated bacteria and tumor-associated markers. Red color is indicating positive correlation; green color is standing for negative correlation whereas black color is showing no correlation. ∗p ≤ 0.05: ∗∗p ≤ 0.01.

Several cachexia-associated bacteria were found down-regulated with Rb3/Rd treatments (Table S5). Especially, the abundances of species from genera *Butyricimonas*, *Campylobacter*, *Dysgonomonas*, *Fusobacterium* and *Helicobacter* were lower in Rb3/Rd treated mice compared to control group (Table S5). Previously, genus *Campylobacter* has been abundantly detected in CRC patient^39^. We detected two species from genus *Campylobacter* (C. *canadensis and C. faecalis*) and both the species were less abundant in Rb3/Rd treated groups. Similarly, *Dysgonomonas* species have been associated with hepatocellular carcinomas and liver abscess^40,41^. Rb3/Rd significantly (p = 0.01) decreased the density of genus *Dysgonomonas* compared to control group. Several studies have reported *Helicobacter* spp. in gut-associated cancer development^42^ and we found that Rb3/Rd substantially deteriorated *Helicobacter* abundance (Table S5). Moreover, most of the taxa from table-S5 were positively correlating with the expression profile of pro-inflammatory/tumor markers (Fig. 8). For instance, several species from genera *Porpphyromonas* (*P. canis*, *P circumdentaria* and *P gulae*), *Helocobacter* (*H. ganmani*, *H. rodentium* and *H. brantae*) and *Dysgonomonas wimpennyi* were positively correlated with pro-inflammatory/tumor markers (Fig. 8).

## Discussion

More than 150 different types of saponins have been identified for anti-cancer properties^43^. However, the major bioactive constituents of saponins are largely unknown. In our previous study, we reported anti-cancer properties of GpS in Apc^Min/+^ mice and xenograft nude mice^21,22^. To determine major anti-cancer compounds of GpS, in this study we are reporting two of the most abundant compounds of GpS for anti-CRC properties in Apc^Min/+^ mice. Treatments of ginsenosides Rb3 and Rd effectively excerted anti-polyposis by holistically improving intestinal microenvironment, mucosal immunity, and GM composition.

During the treatments, we did not observe Rb3/Rd-associated changes in body weight of the mice. Compared to the control group, number and size of the intestinal polyps were significantly reduced in the Rb3/Rd treatment groups, and were comparatively more evident in Rd-treated Apc^Min/+^ mice. Improved intestinal lining with the elevated count of Paneth cells and goblet cell was noticed in Rb3/Rd treated Apc^Min/+^ mice. The α-defensins and lysozyme secreted by Paneth cells, together with mucins secreted by goblet cells form a strong anti-microbial barrier against the invasion of pathogens^44^. To determine the molecular mechanism, arrays of signaling molecules were evaluated for the anti-cancer effect of Rb3/Rd; and suppressed expressions of iNOS, p-STAT3, and p-Src was detected. These signaling molecules are known for promoting CRC and targets for cancer therapy^44^. One of the common features of Apc^Min/+^ mice is the dysbiotic intestinal lining with up-regulated N-cadherin and down-regulated E-cadherin expression. Aberrant expressions of these proteins have been associated with poor intestinal barrier and CRC prognosis^44^. Rb3 and Rd effectively reinstated the expression profile of E-cadherin and N-cadherin in Apc^Min/+^ mice. Normal expression of E-cadherin is also important for maturation of Paneth cells and goblet cells^45^. We suggest that down-regulated p-STATS might account for the altered expression profile of these trans-membrane proteins upon Rb3/Rd treatment, as activation of STAT can negatively regulate E-cadherin and positively modulate N-cadherin^21^ ^, 44^.

Comparatively, Rd clearly promoted IFN-γ and TNF-α expression. IFN-γ is an anti-oncogenic protein that monitors CRC development and contributes to CRC suppression. Recently, Wang *et al*.^46^ identified that colorectal cancer cells propagate in the absence of IFN-γ in Apc^Min/+^ mice. Looking at overall anti-cancer properties of ginsenosides Rd, and in the light of previous report^47^, we assume that TNF-α could contribute to CRC cells apoptosis. Whereas, in contrast to our previous results^21^, we noticed the increasing expression of FOXP3 in Apc^Min/+^ mice in this study. FOXP3 role in CRC development is not defined and discrepancies exist^48^. However, Saito *et al*.^49^ have recently associated higher expression of FOXP3 with poor prognosis in type-A CRC. Moreover, mucosal immunity of Apc^Min/+^ mice substantially remodeled with Rb3/Rd treatments; anti-tumor cytokines were promoted and pro-tumor cytokines were suppressed. For example, Rb3/Rd suppressed IL-23 expression, a pro-inflammatory cytokine that has been predominantly detected in the majority of the tumor types^50^. Rb3/Rd-associated down-regulation of IL-6 could contribute to the suppression of STAT3 through IL-6R signaling^51^. After detecting remodeled mucosal immunity, we were expecting modified GM diversity and composition.

Being in close proximity, mucosal immunity is influencing or being influenced by microbial composition in the gut. To determine Rb3/Rd associated changes in GM composition, initially, ERIC-PCR was performed and detected clear changes in Rb3/Rd treated groups and non-treated Apc^Min/+^ mice. For in-depth analysis, 16S amplicon deep sequencing was employed that indicated dynamic modulations in GM diversity and composition. We observed enriched bacterial diversity in Rb3/Rd treated groups. These results are in agreement with our previous findings^21^. Among the dominant phyla, the abundance and diversity of Proteobacteria significantly reduced with Rb3/Rd treatments. Proteobacteria is one of the main bacterial phyla that reflect GM dysbiosis. This phylum has been frequently associated with inflammation, disturbed mucus layer, and CRC progression^30^; which are the commonly detected feature of the Apc^Min/+^ mice. We believe that Rb3/Rd treatment regulated phylum Proteobacteria by promoting immune regulatory cytokines (IL-10 and IL-4). Previously, the abundance of Proteobacteria has been regulated with IL-10 intervention^52^.

One of the most affected families from phylum Proteobacteria was Helicobacteriaceae that could be related to the lower abundance of *Helicobacter* species. Several *Helicobacter* species have been associated with different types of intestinal cancer^53^. Besides, several other cachexia-associated bacteria (such as *Dysgonomonas* spp., *Campylobacter* spp. and *Mycoplasma* spp.) were down-regulated with Rb3/Rd treatments^39–41,54–58^. Lower abundance of these species was correlated with the lower expression of pro-inflammatory markers (such as iNOS, CXCL10, and IL-1β). In agreement with previous studies^11,59^, we also observed that *Akkermansia muciniphila* and some of the *Blautia* species (e.g. *B. coccoides, B. obeum and B. schinkii*) were associated with pro-inflammatory/tumor markers. *Blautia coccoides* is one of the pathogenic bacteria from genus *Blautia* that decreased with Rb3/Rd treatment. This bacterium has also been abundantly detected in CRC patients^11^. However, the exact mechanism through which Rb3 and Rd suppressed pro-inflammatory markers and down-regulated cancer-associated bacteria is yet to be determined.

In contrast to our previous findings, we did not observe significant changes in class Clostridia diversity and composition^22^. Moreover, family Bacteroidaceae profoundly enriched with Rb3/Rd treatments. This increase could be mainly associated with promoted growth of health-promoting bacteria *Bacteroides acidifaciens* and *Bacteroides xylanisolvens*. Both the species are improving host digestion and metabolism. *Bacteroide sacidifaciens* is known for anti-obesity, anti-metabolic disorders and anti-cancer properties^22^. Whereas, *Bacteroides xylanisolvens* is xylan-degrading bacterium that is dominantly detected in healthy individuals^37^. In addition, we also noticed the enriched abundance of genera *Prevotella* in Rb3/Rd treated mice. *Prevotella* spp. are predominate in mammalian gut and has been proposed for energy extraction from oligosaccharides and resistant starches^60^. The abundance of hydrogenotrophic bacteria (such as *Desulfovibrio piger*, a H_2_S-producing bacterium) promoted with Rb3/Rd treatments^61,62^. H_2_S is normally found in the body and has been suggested for prominent physiological functions^62,63^. Besides, H_2_S has been suggested to be present in more than 90% of neurons in enteric nervous system^63,64^. However, abnormal growth of H_2_S-producing bacteria has been also associated with inflammation and mucosal disruption^63^.

In addition, lactic acid-producing bacteria, such as *Bifidobacterium* spp. and *Lactobacillus* spp., were up-regulated with Rb3/Rd treatments. Several studies have reported anti-CRC properties of these beneficial bacteria^65,66^. Recently, *Bifidobacterium* spp. effectively recovered mucosal barrier in CRC patients^65^. In addition, several *Lactobacillus* spp. are down-regulating CRC by enzymatic detoxification, mucosal protection, and short chain fatty acid production^67^.

## Conclusion

In summary, ginsenosides Rb3 and Rd are two major compounds isolated from *Gynostemma pentaphyllum* that holistically improved gut microenvironment and induced anti-polyposis in Apc^Min/+^ mice. At the molecular level, Rb3/Rd suppressed cancer-promoting signaling markers and improved intestinal barrier by down-regulating pro-inflammatory markers and reinstating altered expression of E-cadherin and N-cadherin. Besides, mucosal immunity and GM composition modified in favor of anti-polyposis. Interestingly, bacterial groups that are previously reported for pro-inflammation were substantially restricted with Rb3/Rd treatments. Whereas, several beneficial bacteria were promoted that may contribute to Rb3/Rd-associated improved health. In conclusion, this study revealed that triterpenoid Rb3 and Rd induced alterations of the commensal gut microbiota that coincided with improvements in gut barrier microenvironment and reduced polyp formation in Apc^Min/+^ mice.

## Materials and Methods

### Animals and treatments

Heterozygous male Apc^Min/+^ (C57BL/6J-*Apc* ^*Min*/+^) mice were purchased from Jackson Laboratory. Mice were housed in a 12-h light–dark cycle facility and were fed with PicoLab®Rodent Diet 20-5053 (LabDiet, USA). All the mice had free access to food and water. Ginsenoside Rb3 (Purity 99.65%) and ginsenoside Rd (Purity 99.23%) were purchased from Man-Si-Te biological technological CO., Ltd (Chengdu, China). Rb3 and Rd were dissolved in 0.5% carboxymethyl cellulose (CMC). Total 32 male Apc^Min/+^ mice (aged 6 weeks) were divided into three groups; 10 mice in the control group and 22 mice equally divided for Rb3 and Rd treatments. The mice  were daily gavage with a single dose of Rb3 or Rd at 20 mg/kg, or solvent control. The treatments were carried out for 8 consecutive weeks. The dosage was chosen based on our previous results and others published reports on the mouse dosages of ginsenosides. American Veterinary Medical Association guidance was followed for carbon dioxide based euthanasia. All the experimental processes were conducted within the approved guidelines of the Ethics Review Committee for Animal Research of Macau University of Science & Technology.

### Fecal samples collection and bacterial genomic DNA extraction

Fecal samples of the individual mouse were collected before treatments and at the end of the experiment (Fig. 1A) according to Lei *et al*., 2016. All the samples were directly stored at −80 °C for later DNA extraction. Total genomic DNA was extracted using QIAamp DNA Stool Mini Kit (QIAGEN) according to the manufacturer’s manual.

### Animal dissection and polyp counting

Four mice per group were dissected for intestinal section collection and polyp counting. 2 cm of the colon and small intestine were separated at the cecal junction, rinsed with PBS and then fixed in 10% formalin for later histological sections. The mucosa was also collected from colon and distal small intestine. The rest portion was cut open longitudinally, rinsed with PBS and fixed in 10% formalin, then stained with methyl blue. The polyps number and sizes were scored under a dissecting microscope.

### Enterobacterial Repetitive Intergenic Consensus (ERIC)-PCR analysis

The extracted fecal DNA was analyzed for highly conserved ERIC regions with a pair of ERIC primers sequences: ERIC 1 (5′-ATGTAAGCTCCTGGGGATTCAC-3′) and ERIC2 (5′-AAGTAAGTGACTGGGGTGAGCG-3′). PCR conditions were set as previously described^22^. Obtained ERIC-PCR products were separated in 2% agarose gel at 100 V for 40 minutes. Bands were visualized with Gel Doc XR+ system and digitized for microbial clustering analysis using SIMCA-P 14.0 tool (Umetrics, Umea, Sweden) with confidence level 95% (p < 0.05).

### 16S rRNA gene pyrosequencing of fecal DNA samples and data analysis

All the metagenomic DNA samples were processed for GM composition using 16S pyrosequencing as previously described^22^. In short, a final PCR reaction volume of 25 µl was comprised of 2.5 µl 10X Expand High Fidelity (EHF) buffer (Roche), 300 nM of each primer (563 F and 1064 R), 0.1–2 µl DNA, 2.6 units of EHF enzyme mix (Roche), 200 µl PCR Grade Nucleotide Mix. Milli-Q H_2_O was used for reaction adjustment. 563 F primer was barcoded with a unique 11 nucleotide tag. The PCR conditions were adjusted as described^22^. PCR product size was analyzed on 1% agarose gel and purified with Gel Extraction Kit (Life Technology). After quantification, Emulsion-PCR and pyrosequencing were performed on the GS Junior System (454 Life Sciences Corp., Branford, CT, USA) following manufacturer guidelines. Raw reads were filtered for high quality using Quantitative Insights into Microbial Ecology (QIIME) version 5.0. Reads with the low quality score (less than 25), shorter size (less than 200 bp) and longer than 1 kb were removed. Additional filters were performed for homopolymers, uncorrectable barcodes, and primers mismatches. High-quality reads were assigned to respective samples and aligned and cluster to operational taxonomic units (OTUs) at 97% identity using NCBI Short Read Archive Database. Metagenomic data of this study is submitted in the NCBI database under the project no. PRJNA355952.

### Histology and immunohistochemistry

H&E staining and immunohistochemical (IHC) staining were carried out with 5 μm thick paraffin sections using standard procedures. Immunohistochemistry was employed with an anti-lysozyme antibody (A0099, DAKO), and LSAB+System-HRP kit (K0679, DAKO). The tissue sections were mounted and viewed under a Leica microscope. Images were taken with a Leica camera (DFC310 FX) and Leica Application Sute software (Version 4.4.0, Switzerland).

### Quantitative reverse transcription polymerase chain reaction (qRT-PCR)

The qRT-PCR were conducted according to our previous study^23^. In brief, mucosal samples were processed for RNA extraction using RNeasy Mini Kit (QIAGEN, Hilden, Germen) followed the manufacturer protocol. RNA was quantified using NanoDrop 2000C spectrophotometer (Thermo, USA). 5 μg of total RNA was used to synthesize the first-strand cDNA using random primers and SuperScript II reverse transcriptase (Invitrogen, Carlsbad, CA, USA). Power SYBR® Green PCR Maser Mix (Applied Biosystems Inc., Carlsbad, CA, USA) was used for PCR reaction. qPCR working conditions were followed as previously described^21^. The qPCR was conducted using Applied Biosystems ViiA™ 7 PCR system (Carlsbad, CA, USA). β-actin was used as the internal control to normalize the PCR reaction of each specific marker. Prior to the PCR analysis, hypoxanthine-guanine phosphoribosyl transferase 1 (Hprt1) was used and compared with β-actin for the expression stability, and the two reference genes showed a similar expression pattern among different samples. We then chose β-actin as the internal control for the rest of the qRT-PCR. The 2^−ΔΔCt^ method was applied to calculate the fold change of relative gene expression. ΔΔCt = (Ct_treatment_target gene_ − Ct_treatment_reference gene_) − (Ct_control_target gene_ − Ct_control_reference gene_).

qRT-PCR was used to measure the RNA expression of cytokines in mucosa, including M1 macrophage marker (iNOS and CXCL 10), M2 macrophage marker (arginase 1, MR, Trem 2, Ym 1), pro-inflammatory cytokines (IL-1β, IL-6, IL-12, IL-17, IL-23), anti-inflammatory cytokines (IL-4, IL-10), and other anti-inflammatory markers (TNF-α, INF-γ and FOXP3). The sequences of the primers used were listed in Table S6. The cytokines were chosen on the basis of their implication in CRC developments^68,69^.

### Western blot analysis

Western blot analysis was carried out with standard methods on the mucosal protein lysates obtained from each mouse. Specific antibodies against p-Stat3 (#9138), Stat3 (#9132), p-Src (#6943), Src (#2123), N-Cadherin (#4061), E-Cadherin (#3195), arginase-1 (#9819), and iNOS (#2982) were purchased from Cell Signaling Technology (Beverly, MA). Anti-FOXP3 antibody (ab54501) was purchased from Abcam (Cambridge, UK) and anti-α-tubulin (sc-20357) was purchased from Santa Cruz Biotechnology (Santa Cruz, CA).

### Statistical analysis

SPSS version 22 was used for statistical analysis. Bacterial taxa data normality was ascertained with Kolmogorov–SmirnovDtest. One-way ANOVA (for parametric data) and Kruskal–Wallis tests (for non-normal data) were performed to observe significantly different bacterial taxa among the groups. Bonferroni and Dunn-Bonferroni tests were respectively performed for parametric and non-parametric multiple comparison p values correction. R packages phyloseq (1.14.0) and VEGAN (2.2-1) were used for alpha and beta diversity analysis^70–72^. Cytoscape (3.4.0) was used for networking analysis. Pearson’s correlation was calculated to explore the relationship between relative abundance of bacteria and modulated expression of tumor-associated markers and the correction was made using Benjamini-Hochberg false discovery rate. Partial least squares discriminant analysis (PLS-DA) was performed to visualize the changes of microbial communities before and after treatments using SIMCA-P 14.0 tool (Umetrics, Umea, Sweden) for which the confidence level was set at 95% (p < 0.05). The rest data was statistically analyzed using one-way ANOVA followed by Dunnett’s post hoc test with the GraphPad Prism version 5.00 (GraphPad Software, San Diego, CA, USA) or Student’s t-test at p < 0.001(***), p < 0.01(**) or p < 0.05(*).

### Accession numbers

The data discussed in this manuscript have been deposited in SRA NCBI and accesable through project no. PRJNA355952.

## Electronic supplementary material

Supplementary file
