# Supplementary material for: Ginsenosides Rb3 and Rd reduce polyps formation while reinstate the dysbiotic gut microbiota and the intestinal microenvironment in ApcMin/+ mice
Source: Sci Rep. 2017 Oct 2;7:12552. doi: 10.1038/s41598-017-12644-5 (PMC5624945; doi:10.1038/s41598-017-12644-5)
Supplement: Supplementary file 1 — Supplementary file [file 41598_2017_12644_MOESM1_ESM.pdf]

Ginsenosides Rb3 and Rd reduce polyps formation while reinstate the dysbiotic gut microbiota and the intestinal microenvironment in $Apc^{Min/+}$ mice

Guoxin Huang,[a#] Imran Khan,[a#] Xiaoang Li,[a] Lei Chen,[c] Waikit Leong,[a] Leung Tsun Ho,[b] W.L. Wendy Hsiao[a]*

[a]State Key Laboratory of Quality Research in Chinese Medicine, Macau University of Science and Technology, Macau, China

[b]Department of Pathology, University Hospital, Macau University of Science and Technology, Macau, China

[c]Department of Genetics, Rutgers University, New Brunswick, USA

[#]: These persons made equal contribution to this manuscript.

*corresponding author: W.L. Wendy Hsiao

Email: wlhsiao@mus.edu.mo

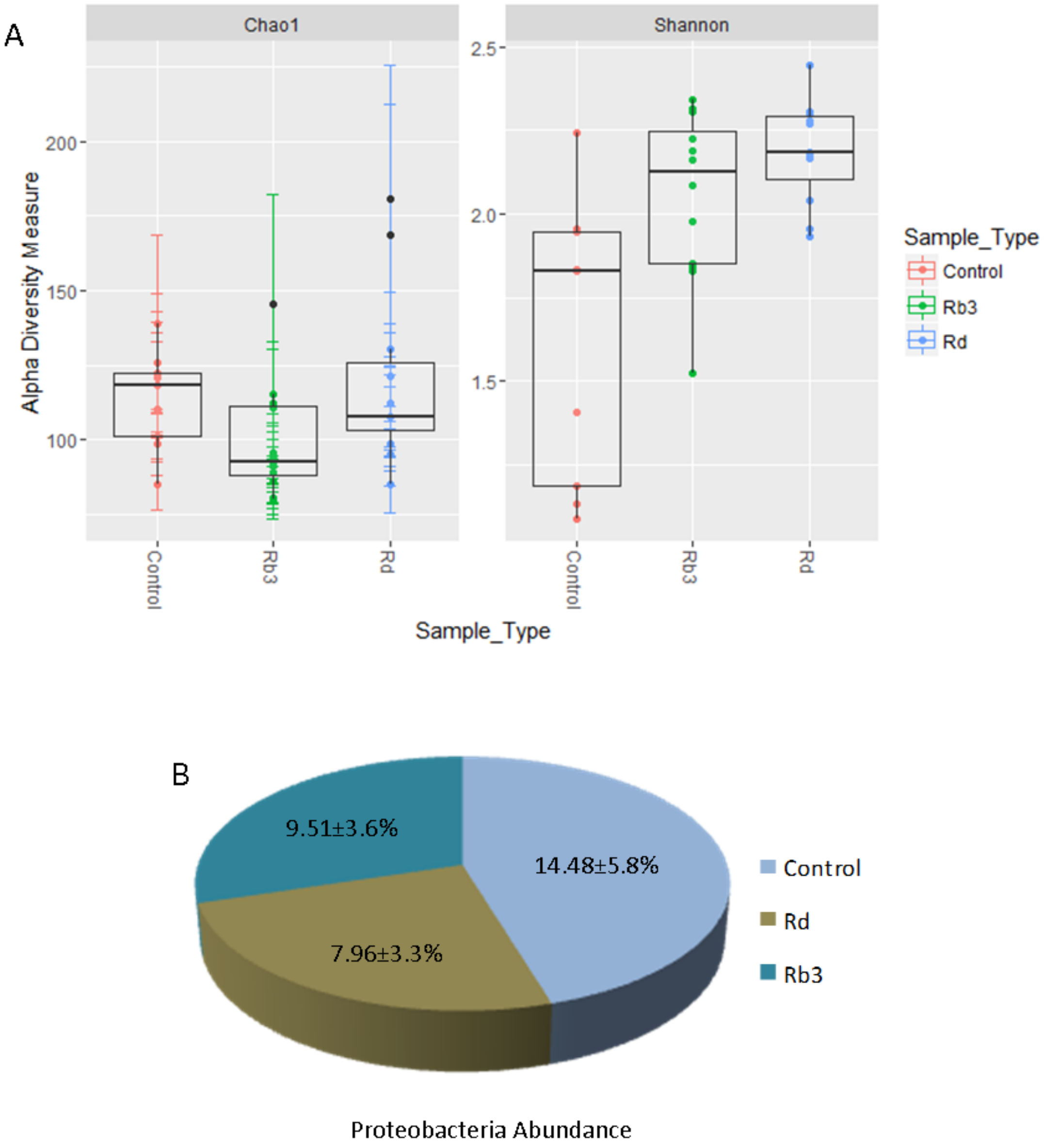

Figure S1. Alpha diversity analysis of phylum Proteobacteria. (A) The dataset was sub-group to Proteobacteria and OTUs diversity analyses were performed. (B) Average relative abundance of phylum Proteobacteria.

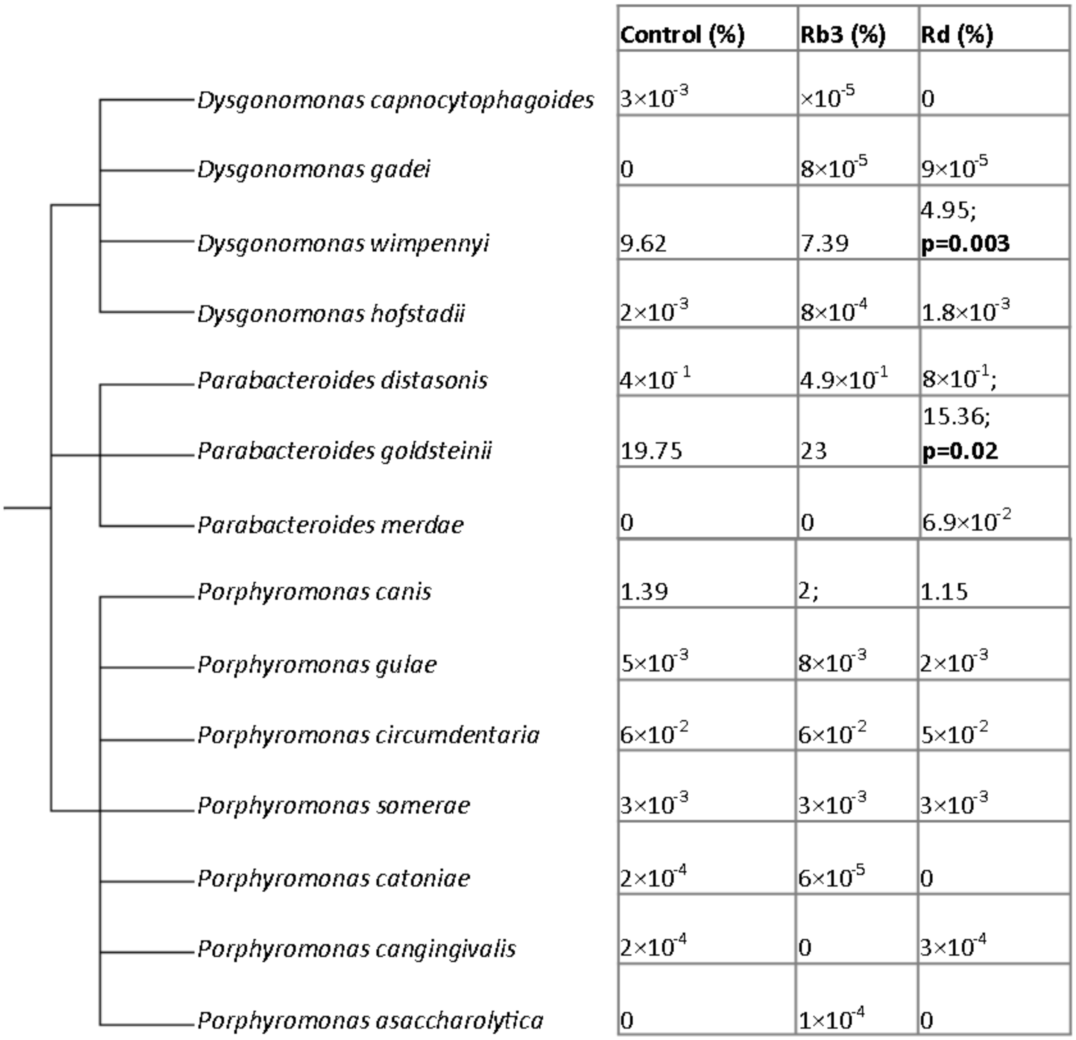

Species	Control (%)	Rb3 (%)	Rd (%)
Dysgonomonas capnocytophagoides	3×10^{-3}	$\times10^{-5}$	0
Dysgonomonas gadei	0	8×10^{-5}	9×10^{-5}
Dysgonomonas wimpennyi	9.62	7.39	4.95; **p=0.003**
Dysgonomonas hofstadii	2×10^{-3}	8×10^{-4}	1.8×10^{-3}
Parabacteroides distasonis	4×10^{-1}	4.9×10^{-1}	8×10^{-1};
Parabacteroides goldsteinii	19.75	23	15.36; **p=0.02**
Parabacteroides merdae	0	0	6.9×10^{-2}
Porphyromonas canis	1.39	2;	1.15
Porphyromonas gulae	5×10^{-3}	8×10^{-3}	2×10^{-3}
Porphyromonas circumdentaria	6×10^{-2}	6×10^{-2}	5×10^{-2}
Porphyromonas somerae	3×10^{-3}	3×10^{-3}	3×10^{-3}
Porphyromonas catoniae	2×10^{-4}	6×10^{-5}	0
Porphyromonas cangingivalis	2×10^{-4}	0	3×10^{-4}
Porphyromonas asaccharolytica	0	1×10^{-4}	0

Figure S2. Phylogenetic tree of species detected in family Porphyromonadaceae. Values are showing average relative abundance of each species. P values are showing statistical change in the relative abundance. Tree was generated using NCBI phyloT.

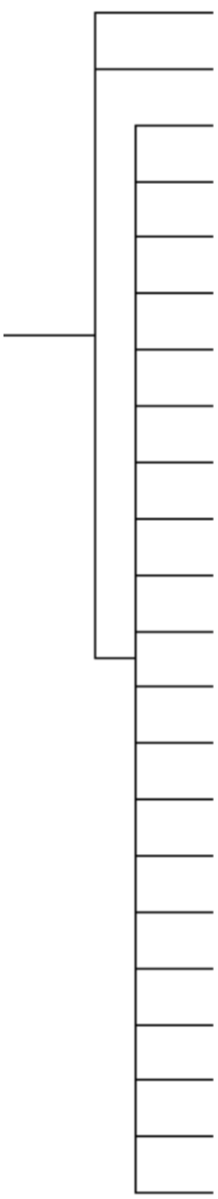

Species	Control (%)	Rb3 (%)	Rd (%)
Paraprevotella clara	76×10^{-2}	12×10^{-1}	37×10^{-1}; **p=0.007**
Prevotella tannerae	1×10^{-4}	0	0
Prevotella enoeca	0	0	1×10^{-4}
Prevotella amnii	4×10^{-4}	1×10^{-3}; **p=0.04**	8×10^{-4}
Prevotella aurantiaca	6×10^{-4}	4×10^{-4}	6×10^{-4}
Prevotella loescheii	3.6×10^{-1}	7×10^{-1}	9.1×10^{-1}
Prevotella buccalis	3×10^{-3}	5×10^{-3}; **p=0.047**	5.3×10^{-3}
Prevotella maculosa	4.4×10^{-2}	6×10^{-2}	9.9×10^{-2};
Prevotella timonensis	1×10^{-4}	5×10^{-4}	3×10^{-4}
Prevotella multisaccharivorax	1×10^{-3}	1×10^{-3}	1.4×10^{-3}
Prevotella paludivivens	4×10^{-2}	$\times10^{-2}$	5.9×10^{-2}; **p=0.007**
Prevotella dentalis	4×10^{-3}	6×10^{-3}	6.5×10^{-3}
Prevotella marshii	5×10^{-3}	3×10^{-3}	5×10^{-3}
Prevotella dentasini	20.5	17.93	23.8
Prevotella pleuritidis	1×10^{-3}	1×10^{-3}	1.8×10^{-3}
Prevotella albensis	3×10^{-2}	2.3×10^{-2}	3.3×10^{-2};
Prevotella multiformis	8×10^{-3}	7×10^{-3}	8×10^{-3}
Prevotella micans	1×10^{-3}	4×10^{-3}	3.7×10^{-3}
Prevotella saccharolytica	0	4×10^{-4}	0
Prevotella shahii	3×10^{-2}	3×10^{-2}	3.6×10^{-2}
Prevotella corporis	0	0	1×10^{-4}
Prevotella baroniae	0	0	1×10^{-4}

Figure S3. Phylogenetic tree of species detected in family Prevotellaceae. Values are showing average relative abundance of each species. p values are showing statistical change in the relative abundance. Tree was generated using NCBI PhyloT.

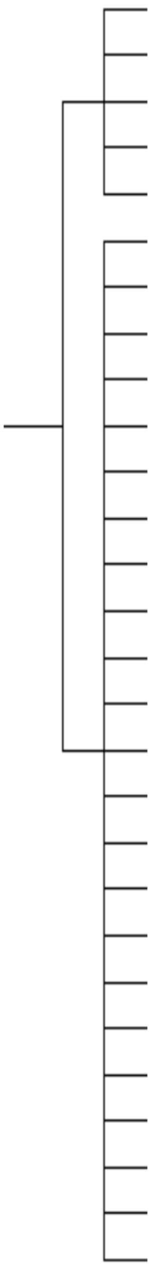

Species	Control (%)	Rb3 (%)	Rd (%)
Parabacteroides merdae	0	0	7×10^{-2}
Parabacteroides johnsonii	5×10^{-2}	6×10^{-2}	4×10^{-2}
Bacteroides chinchillae	1×10^{-4}	1×10^{-4}	0
Parabacteroides distasonis	4.3×10^{-1}	4.9×10^{-1}	8×10^{-1}; **p=0.04**
Parabacteroides goldsteinii	19.74	23.02	15.35; **p=0.02**
Bacteroides vulgatus	1×10^{-4}	0	7×10^{-2}
Bacteroides gallinarum	6×10^{-4}	2×10^{-3}; **p=0.04**	4×10^{-3}; **p=0.01**
Bacteroides salanitronis	2.5×10^{-3}	2×10^{-3}	1×10^{-3}; **p=0.002**
Bacteroides coprocola	1×10^{-4}	0	0
Bacteroides stercorirosoris	2.8×10^{-2}	1.5×10^{-1}	1.5×10^{-1};**p=0.003**
Bacteroides denticanum	1.8	2.3	2.4; **p=0.01**
Bacteroides cellulosilyticus	2×10^{-2}	3.8×10^{-1}	3.7×10^{-1}; **p=0.007**
Bacteroides paurosaccharolyticus	5.06	5.67	5.93
Bacteroides thetaiotaomicron	1×10^{-4}	0	3×10^{-4}
Bacteroides oleiciplenus	1×10^{-4}	2×10^{-3}; **p=0.018**	2×10^{-3}
Bacteroides stercoris	0	9×10^{-5}	9×10^{-5}
Bacteroides graminisolvens	7.5×10^{-2}	1.5×10^{-1}; **p=0.004**	2.6×10^{-1}
Bacteroides rodentium	3×10^{-1}	5.1×10^{-1}	2.9×10^{-1}
Bacteroides zoogleoformans	6×10^{-4}	1×10^{-3}	1×10^{-3}
Bacteroides intestinalis	4.9×10^{-3}	7×10^{-2}; **p=0.001**	7×10^{-2}; **p=0.01**
Bacteroides dorei	0	0	8×10^{-5}
Bacteroides uniformis	1×10^{-4}	0	0
Bacteroides nordii	2×10^{-4}	1×10^{-3}	1×10^{-3}; **p=0.001**
Bacteroides heparinolyticus	9×10^{-4}	1×10^{-3}	2×10^{-3}; **p=0.04**
Bacteroides xylanisolvens	5.9×10^{-1}	1.4; **p=0.04**	2.3±0.9
Bacteroides acidifaciens	1.13	2.3; **p=0.002**	4.43
Bacteroides helcogenes	0	0	1.7×10^{-4}
Bacteroides sartorii	2.8×10^{-1}	1.6×10^{-1}; **p=0.04**	2.4×10^{-2}

Figure S4. Phylogenetic tree of species detected in family Bacteroidaceae. Values are showing average relative abundance of each species. p values are showing statistical change in the relative abundance. Tree was generated using NCBI PhyloT.

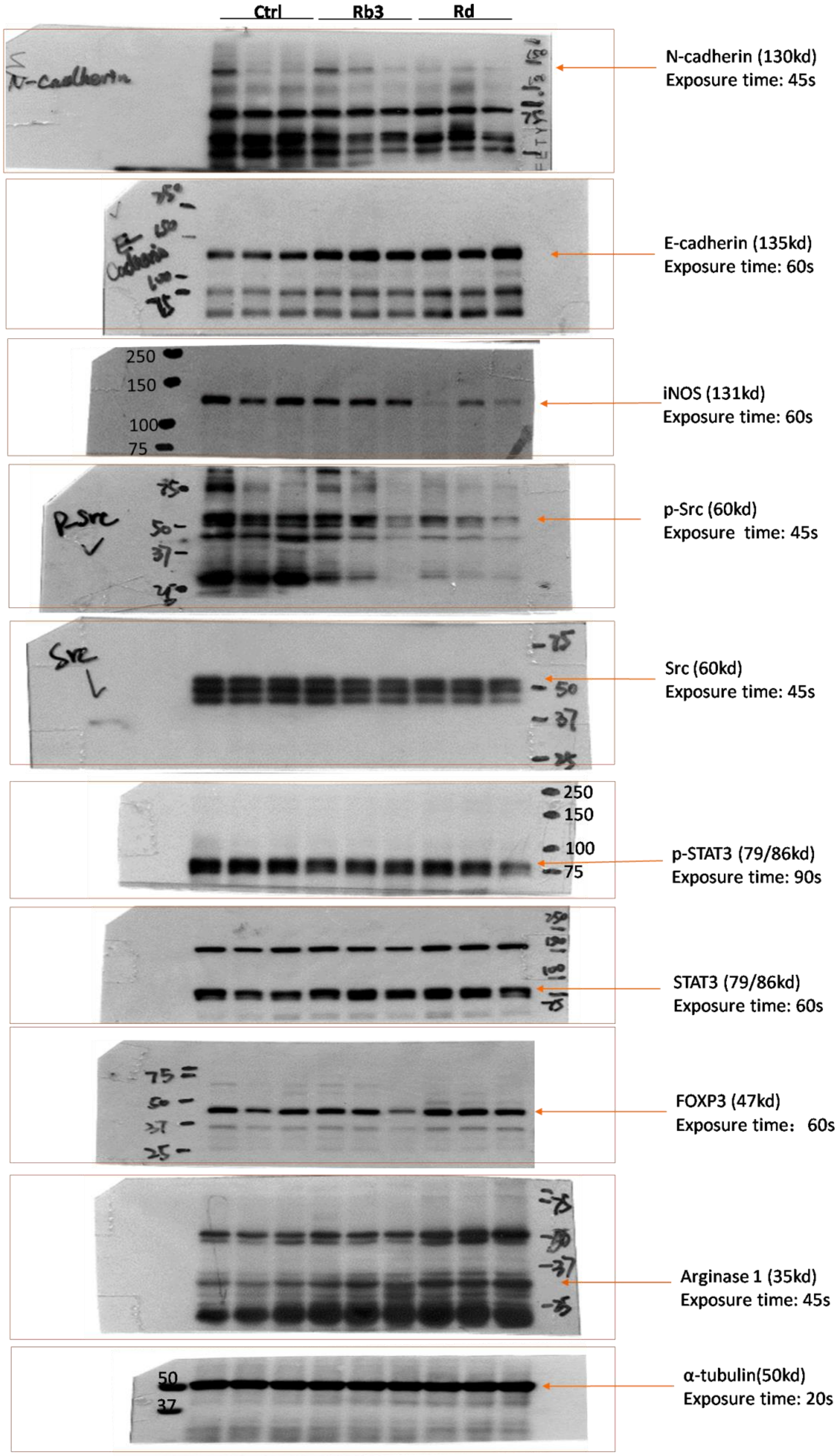

Figure S5. The full-length blots of the Western blots showed in Figure 2. Each lane represents sample obtained from individual mouse (n=3).

Table S1. List of unique species from three dominant phyla detected in the control group

Species	Phyla	Relative abundance (%)
Myroides injenensis	Bacteroidetes	1×10^{-4}
Balneola vulgaris	Bacteroidetes	8.49×10^{-5}
Bacteroides coprocola	Bacteroidetes	8.39×10^{-5}
Bacteroides uniformis	Bacteroidetes	7.67×10^{-5}
Prevotella tannerae	Bacteroidetes	7.14×10^{-5}
Natronincola peptidivorans	Firmicutes	4.8×10^{-4}
Lactobacillus faeni	Firmicutes	1×10^{-4}
Bacillus okhensis	Firmicutes	1×10^{-4}
Paenibacillus donghaensis	Firmicutes	9.7×10^{-5}
Salinicoccus luteus	Firmicutes	9.7×10^{-5}
Thermobacillus xylanilyticus	Firmicutes	9.7×10^{-5}
Paenibacillus ourofinensis	Firmicutes	8.49×10^{-5}
Anoxybacillus tengchongensis	Firmicutes	8.49×10^{-5}
Salinicoccus iranensis	Firmicutes	7.94×10^{-5}
Coprothermobacter platensis	Firmicutes	7.94×10^{-5}
Jeotgalicoccus halotolerans	Firmicutes	7.67×10^{-5}
Jeotgalicoccus psychrophilus	Firmicutes	7.67×10^{-5}
Thauera selenatis	Proteobacteria	1.9×10^{-4}
Mesorhizobium huakuii	Proteobacteria	1.7×10^{-4}
Acinetobacter junii	Proteobacteria	1.6×10^{-4}
Acinetobacter gerneri	Proteobacteria	1×10^{-4}
Desulfovibrio longreachensis	Proteobacteria	1×10^{-4}
Phenylobacterium mobile	Proteobacteria	9.71×10^{-5}
Bradyrhizobium pachyrhizi	Proteobacteria	9.71×10^{-5}
Agrobacterium rubi	Proteobacteria	9.71×10^{-5}
Helicobacter winghamensis	Proteobacteria	9.71×10^{-5}
Lysobacter deserti	Proteobacteria	8.49×10^{-5}
Ralstonia detusculanense	Proteobacteria	8.39×10^{-5}
Halomonas gudaonensis	Proteobacteria	8.39×10^{-5}
Stenotrophomonas terrae	Proteobacteria	8.39×10^{-5}
Lautropia mirabilis	Proteobacteria	8.16×10^{-5}
Moritella japonica	Proteobacteria	7.67×10^{-5}
Vibrio litoralis	Proteobacteria	7.67×10^{-5}
Gluconobacter morbifer	Proteobacteria	7.67×10^{-5}
Brevundimonas olei	Proteobacteria	7.3×10^{-5}

Table S2. List of unique species from three dominant phyla detected in the Rb3 group

Species	Phyla	Relative abundance (%)
Lentibacillus kapialis	Firmicutes	8×10^{-5}
Virgibacillus byunsanensis	Firmicutes	7×10^{-5}
Bacillus pseudofirmus	Firmicutes	8×10^{-5}
Lactobacillus intestinalis	Firmicutes	7×10^{-4}
Lactobacillus panis	Firmicutes	9×10^{-5}
Lactobacillus oris	Firmicutes	9×10^{-5}
Ureibacillus thermophilus	Firmicutes	1×10^{-4}
Staphylococcus intermedius	Firmicutes	7×10^{-5}
Caloramator viterbiensis	Firmicutes	8×10^{-5}
Caloramator fervidus	Firmicutes	5×10^{-5}
Clostridium papyrosolvens	Firmicutes	2×10^{-4}
Fusibacter paucivorans	Firmicutes	7×10^{-5}
Helcococcus ovis	Firmicutes	7×10^{-5}
Mogibacterium timidum	Firmicutes	1×10^{-4}
Anaerofilum agile	Firmicutes	1×10^{-4}
Syntrophomonas bryantii	Firmicutes	5×10^{-5}
Porphyromonas asaccharolytica	Bacteroidetes	1×10^{-4}
Prevotella saccharolytica	Bacteroidetes	3×10^{-4}
Chryseobacterium culicis	Bacteroidetes	2.7×10^{-4}
Exiguobacterium taiwanense	Bacteroidetes	7×10^{-5}
Flavobacterium weaverense	Bacteroidetes	8×10^{-5}
Polaribacter filamentus	Bacteroidetes	1×10^{-4}
Tenacibaculum japonica	Bacteroidetes	2×10^{-4}
Lewinella marina	Bacteroidetes	2×10^{-4}
Ochrobactrum thiophenivorans	Proteobacteria	7×10^{-5}
Kaistia soli	Proteobacteria	8×10^{-5}
Novispirillum peregrinum	Proteobacteria	4×10^{-4}
Novosphingobium yangbajingensis	Proteobacteria	1×10^{-4}
Novosphingobium acidiphilum	Proteobacteria	5×10^{-5}
Sphingomonas roseiflava	Proteobacteria	2.8×10^{-4}
Ancylobacter rudongensis	Proteobacteria	8×10^{-5}
Burkholderia ubonensis	Proteobacteria	7×10^{-5}
Desulfitobacterium chlororespirans	Proteobacteria	2.9×10^{-5}
Desulfovermiculus halophilus	Proteobacteria	9×10^{-5}
Desulfovibrio gracilis	Proteobacteria	7×10^{-5}
Syntrophobacter wolinii	Proteobacteria	8×10^{-5}
Helicobacter canis	Proteobacteria	8×10^{-5}

Microbulbifer epialgicus	Proteobacteria	9×10^{-5}
Morganella psychrotolerans	Proteobacteria	9×10^{-5}
Serratia ureilytica	Proteobacteria	9×10^{-5}
Moritella yayanosii	Proteobacteria	9×10^{-5}
Chelonobacter oris	Proteobacteria	9×10^{-5}

Table S3. List of unique species from three dominant phyla detected in the Rd group

Species	Phyla	Relative abundance (%)
Bacteroides dorei	Bacteroidetes	8×10^{-5}
Bacteroides helcogenes	Bacteroidetes	1×10^{-4}
Parabacteroides merdae	Bacteroidetes	6.9×10^{-2}
Prevotella baroniae	Bacteroidetes	9×10^{-5}
Prevotella corporis	Bacteroidetes	9×10^{-5}
Prevotella enoeca	Bacteroidetes	8×10^{-5}
Chryseobacterium oranimense	Bacteroidetes	1.8×10^{-4}
Maribacter goseongensis	Bacteroidetes	1.7×10^{-4}
Polaribacter dokdonensis	Bacteroidetes	1.9×10^{-4}
Salegentibacter holothuriorum	Bacteroidetes	9×10^{-5}
Alkalibacillus salilacus	Firmicutes	9×10^{-5}
Bacillus horneckiae	Firmicutes	8×10^{-5}
Enterococcus faecalis	Firmicutes	3.2×10^{-4}
Geobacillus thermoglucosidans	Firmicutes	2.6×10^{-4}
Lactobacillus thailandensis	Firmicutes	1.6×10^{-4}
Paenibacillus contaminans	Firmicutes	8×10^{-5}
Streptococcus pluranimalium	Firmicutes	1.8×10^{-4}
Streptococcus thoraltensis	Firmicutes	8×10^{-5}
Weissella minor	Firmicutes	8×10^{-5}
Weissella thailandensis	Firmicutes	8×10^{-5}
Clostridium chromoreductans	Firmicutes	8×10^{-5}
Clostridium frigidicarnis	Firmicutes	9×10^{-5}
Moorella glycerini	Firmicutes	2.4×10^{-4}
Ruminococcus torques	Firmicutes	8×10^{-5}
Thermoanaerobacter sulfurigignens	Firmicutes	8×10^{-5}
Erysipelothrix inopinata	Firmicutes	3.5×10^{-4}
Acidiphilium symbioticum	Proteobacteria	9×10^{-5}
Azospirillum halopraeferens	Proteobacteria	9×10^{-5}
Azospirillum rugosum	Proteobacteria	8×10^{-5}
Devosia terrae	Proteobacteria	8×10^{-5}
Methylobacterium jeotgali	Proteobacteria	8×10^{-5}
Sphingomonas insulae	Proteobacteria	9×10^{-5}
Sphingomonas pseudosanguinis	Proteobacteria	8×10^{-5}
Thalassospira xianhensis	Proteobacteria	9×10^{-5}
Cupriavidus pauculus	Proteobacteria	1.6×10^{-5}
Hydrogenophilus hirschii	Proteobacteria	8×10^{-5}
Variovorax boronicumulans	Proteobacteria	8×10^{-5}
Desulfosarcina ovata	Proteobacteria	1.9×10^{-4}
Desulfovibrio vulgaris	Proteobacteria	8×10^{-5}

Nitrospina gracilis	Proteobacteria	9×10^{-5}
Caminibacter profundus	Proteobacteria	8×10^{-5}
Allochromatium palmeri	Proteobacteria	8×10^{-5}
Amphritea atlantica	Proteobacteria	9×10^{-5}
Enterobacter aceae	Proteobacteria	8×10^{-5}
Halomonas fontilapidosi	Proteobacteria	2.1×10^{-4}
Moraxella caviae	Proteobacteria	2.6×10^{-4}
Pseudomonas panacis	Proteobacteria	8×10^{-5}
Psychrobacter phenylpyruvicus	Proteobacteria	9×10^{-5}
Stenotrophomonas retroflexus	Proteobacteria	8×10^{-5}
Thiothrix nivea	Proteobacteria	2.7×10^{-4}
Vibrio pommerensis	Proteobacteria	8×10^{-5}

Table S4. List of metabolically important bacterial species that modulated with Rb3/Rd treatment

Species	Control (%)	Rb3 (%)	Rd (%)	Percent change in Rb3	Percent change in Rd
Akkermansia muciniphila	0.165	0.025	0.035	-84.84	-78.8
Aminiphilus circumscriptus	0.011	0.018	0.019	66.46	72.92
Bacteroides acidifaciens	1.132	2.343	4.437	106.98	292.031
Bacteroides cellulosilyticus	0.020	0.383	0.374	1789.92	1744.83
Bacteroides graminisolvens	0.075	0.154	0.266	104.53	253.18
Bacteroides intestinalis	0.005	0.070	0.071	1332.15	1355.35
Bacteroides paurosaccharolyticus	5.066	5.671	5.933	11.95	17.11
Bacteroides rodentium	0.301	0.519	0.298	72.55	-0.89
Bacteroides sartorii	0.282	0.169	0.024	-39.99	-91.59
Bacteroides stercorirosoris	0.028	0.151	0.152	437.71	444.31
Bacteroides xylanisolvens	0.600	1.408	2.321	134.88	287.11
Bifidobacterium choerinum	0.033	0.118	0.055	252.87	63.96
Blautia coccoides	2.844	1.806	1.799	-36.5	-36.74
Blautia hansenii	0.707	0.528	0.769	-25.33	8.86
Blautia hydrogenotrophica	0.008	0.016	0.003	97.52	-64.53
Blautia wexlerae	0.911	0.676	0.914	-25.8	0.2564
Butyricimonas synergistica	1.042	0.654	0.384	-37.22	-63.096
Butyricimonas virosa	0.142	0.195	0.172	37.86	21.713
Butyrivibrio proteoclasticus	0.129	0.138	0.205	7.033	58.66
Deferribacter autotrophicus	0.003	0.003	0.177	1.24	5178.36
Desulfonauticus autotrophicus	0.032	0.035	0.052	9.51	63.62
Desulfotomaculum indicum	0.062	0.083	0.055	33.76	-11.98
Desulfovibrio fairfieldensis	0.159	0.117	0.156	-26.4	-1.88
Desulfovibrio litoralis	0.004	0.138	0.041	3269.7	897.27
Desulfovibrio oryzae	0.007	0.011	0.009	63.4	28.86
Desulfovibrio piger	1.266	1.773	1.561	40.03	23.28
Desulfovibrio psychrotolerans	0.202	0.245	0.246	21.02	21.51
Desulfovibrio simplex	0.011	0.015	0.056	35.36	402.6
Desulfurispirillum alkaliphilum	0.015	0.018	0.013	17.58	-12.33
Desulfurispora thermophila	0.064	0.118	0.013	82.98	-80
Dysgonomonas wimpennyi	9.620	7.391	4.951	-23.16	-48.53
Eggerthella sinensis	0.009	0.029	0.015	216.99	62.46
Halanaerobium alcaliphilum	0.041	0.056	0.068	34.81	65.64
Lachnospira pectinoschiza	0.698	0.641	0.540	-8.07	-22.57

Lactobacillus antri	0.002	0.048	0.012	2847.4	660.6
Lactobacillus equi	0.003	0.011	0.019	308.05	635.76
Lactobacillus hayakitensis	0.043	0.166	0.329	290.68	672.37
Lactobacillus johnsonii	0.124	0.115	0.149	-7.48	20.25
Lactobacillus siliginis	0.004	0.017	0.025	352.23	543.5
Lactobacillus taiwanensis	0.024	0.028	0.034	14.68	39.06
Lactobacillus vaginalis	0.001	0.022	0.004	2327.96	366.1
Lentibacillus salinarum	0.015	0.016	0.016	4.27	8.08
Limnobacter litoralis	0.145	0.317	0.551	118.92	281.27
Moryella indoligenes	0.092	0.031	0.036	-66.29	-61.44
Oscillospira eae	2.182	2.519	2.924	15.47	34.05
Oscillospira guilliermondii	0.852	0.759	0.779	-10.89	-8.54
Parabacteroides distasonis	0.438	0.491	0.805	12.12	83.58
Parabacteroides goldsteinii	19.748	23.024	15.356	16.58	-22.24
Parabacteroides johnsonii	0.052	0.062	0.044	19.39	-14.85
Paraprevotella clara	0.756	1.224	3.721	61.77	391.86
Pedobacter kwangyangensis	0.394	0.561	0.350	42.24	-11.27
Peptoniphilus gorbachii	0.018	0.039	0.005	123.58	-71.32
Peptoniphilus methioninivorax	0.018	0.043	0.055	133.46	203.48
Porphyromonas circumdentaria	0.063	0.062	0.055	-1.87	-12.99
Prevotella albensis	0.027	0.029	0.033	6.26	21.91
Prevotella dentasini	20.501	17.935	23.771	-12.52	15.94
Prevotella loescheii	0.361	0.770	0.914	113.49	153.29
Prevotella maculosa	0.044	0.063	0.100	44.927	128.53
Prevotella paludivivens	0.044	0.042	0.059	-2.69	35.74
Prevotella shahii	0.028	0.032	0.037	14.2	29.24
Rhodococcus percolatus	0.062	0.089	0.087	43.03	39.3
Rikenella microfusus	0.860	1.424	0.463	65.57	-46.19
Ruminococcus callidus	0.005	0.016	0.006	246.42	42.04
Ruminococcus flavefaciens	0.032	0.054	0.038	70.98	20.75
Ruminococcus gnavus	0.099	0.260	0.090	163.02	-9.16
Runella limosa	0.019	0.028	0.024	47.92	23.55
Sedimentibacter hydroxybenzoicus	0.919	0.975	0.707	6.13	-23.05
Sutterella sanguinus	0.070	0.653	0.027	831.8	-61.8
Thermodesulfatator atlanticus	0.010	0.012	0.007	26.506	-25.64
Thermodesulfovibrio thiophilus	0.031	0.032	0.029	2.14	-7.85
Thiohalorhabdus denitrificans	0.025	0.025	0.037	1.473	48.56
Tindallia magadiensis	0.026	0.043	0.055	63.39	111.07

Turicibacter sanguinis	0.0002	0.04	0.0003	21099.03	38.43

Table S5. List of cachexia associated bacterial species that reduced with Rb3/Rd treatment

Species	Control (%)	Rb3 (%)	Rd (%)	Percent change in Rb3	Percent change in Rd
Butyricimonas synergistica	1.0416	0.6539	0.3844	-37.22	-63.10
Butyricimonas virosa	0.1416	0.1952	0.1723	37.87	21.71
Campylobacter canadensis	0.0282	0.0272	0.0256	-3.37	-9.25
Campylobacter faecalis	0.0083	0.0040	0.0026	-51.42	-68.84
Dysgonomonas capnocytophagoides	0.0003	0.0001	0	-71.03	-100.00
Dysgonomonas hofstadii	0.0002	0.0001	0.0002	-64.64	-27.63
Dysgonomonas wimpennyi	9.6202	7.3915	4.95	-23.17	-48.53
Fusobacterium naviforme	0.0014	0.0011	0.0021	-20.78	53.50
Helicobacter acinonychis	0.0001	0	0.0001	-100.00	13.38
Helicobacter anseris	0.0002	0.0006	0.0001	222.31	-56.75
Helicobacter baculiformis	0.0007	0.0006	0.0003	-16.18	-50.74
Helicobacter brantae	0.0011	0.0002	0.0003	-85.51	-77.04
Helicobacter canadensis	0.0001	0	0.0001	-100.00	-3.32
Helicobacter cholecystus	0.0001	0	0.0001	-100.00	3.70
Helicobacter equorum	0.0007	0.0003	0.0002	-62.08	-75.23
Helicobacter ganmani	8.3468	0.7313	0.5761	-91.24	-93.10
Helicobacter mastomyrinus	1.7704	2.5918	1.3424	46.39	-24.18
Helicobacter rappini	0.0366	0.0279	0.0212	-23.90	-42.06
Helicobacter rodentium	0.0153	0.0010	0.0003	-93.31	-97.76
Helicobacter salomonis	0.0002	0	0.0001	-100.00	-44.44
Helicobacter suis	0.0002	0	0.0003	-100.00	47.15
Helicobacter suncus	0.4553	0.3365	0.2098	-26.10	-53.93
Helicobacter trogontum	0.0025	0.0012	0.0008	-50.33	-68.86
Helicobacter winghamensis	0.0001	0	0	-100.00	-100.00
Porphyromonas cangingivalis	0.0003	0.0000	0.0003	-100.00	1.18
Porphyromonas canis	1.3956	2.0155	1.1550	44.42	-17.24
Porphyromonas cansulci	0.0003	0.0005	0.0001	72.90	-71.62
Porphyromonas catoniae	0.0002	0.0001	0	-76.17	-100.00
Porphyromonas circumdentaria	0.0633	0.0621	0.0551	-1.87	-13.00
Porphyromonas gulae	0.0052	0.0079	0.0017	51.67	-66.36
Streptococcus bovis	0.0041	0.0023	0.0043	-43.55	5.17

Table S6: List of primers used in qPCR

Target gene	Nucleotide sequence of primer (5' to 3')	
	**Forward**	**Reverse**
β-actin	TGTTACCAACTGGGACGACA	CTGGGTCATCTTTTCACGGT
iNOS	GTTCTCAGCCCAACAATACAAGA	GTGGACGGGTCGATGTCAC
CXCL 10	CCAAGTGCTGCCGTCATTTTC	GGCTCGCAGGGATGATTTCAA
Arginase 1	TGGCTTGCGAGACGTAGAC	GCTCAGGTGAATCGGCCTTTT
MR	GCTGAATCCCAGAAATTCCGC	ATCACAGGCATACAGGGTGAC
Trem 2	CTGGAACCGTCACCATCACTC	CGAAACTCGATGACTCCTCGG
Ym 1	TTATCCTGAGTGACCCTTCTAAG	TCATTACCCTGATAGGCATAGG
IL-1β	GCTGAAAGCTCTCCACCTCA	GGCCACAGGTATTTTGTCGT
IL-6	CTTCCATCCAGTTGCCTTCTTG	AATTAAGCCTCCGACTTGTGAAG
IL-12	ACTCTGCGCCAGAAACCTC	CACCCTGTTGATGGTCACGAC
IL-17	TTTAACTCCCTTGGCGCAAAA	CTTTCCCTCCGCATTGACAC
IL-23	GCACCTGCTTGACTCTGACA	ATCCTCTGGCTGGAGGAGTT
IL-4	GGTCTCAACCCCCAGCTAGT	GCCGATGATCTCTCTCAAGTGAT
IL-10	GCTCTTACTGACTGGCATGAG	CGCAGCTCTAGGAGCATGTG
TNF-α	CAAATGGCCTCCCTCTCAT	CTCCTCCACTTGGTGGTTTG
INF-γ	ATGAACGCTACACACTGCATC	CCATCCTTTTGCCAGTTCCTC
FOXP3	CCCATCCCCAGGAGTCTTG	ACCATGACTAGGGGCACTGTA
